# The regulation of MFG‐E8 on the mitophagy in diabetic sarcopenia via the HSPA1L‐Parkin pathway and the effect of D‐pinitol

**DOI:** 10.1002/jcsm.13459

**Published:** 2024-03-29

**Authors:** Wenqian Zhao, Bin Zhao, Xinyue Meng, Baoying Li, Yajuan Wang, Fei Yu, Chunli Fu, Xin Yu, Xiaoli Li, Chaochao Dai, Jie Wang, Haiqing Gao, Mei Cheng

**Affiliations:** ^1^ Department of Geriatric Medicine, Qilu Hospital, Cheeloo College of Medicine Shandong University Jinan China; ^2^ Key Laboratory of Cardiovascular Proteomics of Shandong Province, Qilu Hospital, Cheeloo College of Medicine Shandong University Jinan China; ^3^ Jinan Clinical Research Center for Geriatric Medicine (202132001) Jinan China; ^4^ Postdoctoral Research Station Shandong University of Traditional Chinese Medicine Jinan China; ^5^ Health Management Center (East Area) Qilu Hospital of Shandong University Jinan China; ^6^ Department of Pharmacy Qilu Hospital of Shandong University Jinan China

**Keywords:** D‐pinitol, HSPA1L, MFG‐E8, Mitophagy, Parkin, Sarcopenia

## Abstract

**Background:**

Diabetic sarcopenia is a disease‐related skeletal muscle disorder that causes progressive symptoms. The complete understanding of its pathogenesis is yet to be unravelled, which makes it difficult to develop effective therapeutic strategies. This study investigates how MFG‐E8 affects mitophagy and the protective role of D‐pinitol (DP) in diabetic sarcopenia.

**Methods:**

In vivo, streptozotocin‐induced diabetic SAM‐R1 (STZ‐R1) and SAM‐P8 (STZ‐P8) mice (16‐week‐old) were used, and STZ‐P8 mice were administrated of DP (150 mg/kg per day) for 6 weeks. Gastrocnemius muscles were harvested for histological analysis including transmission electron microscopy. Proteins were evaluated via immunohistochemistry (IHC), immunofluorescence (IF), and western blotting (WB) assay. In vitro, advanced glycation end products (AGEs) induced diabetic and D‐galactose (DG) induced senescent C2C12 models were established and received DP, MFG‐E8 plasmid (Mover)/siRNA (MsiRNA), or 3‐MA/Torin‐1 intervention. Proteins were evaluated by IF and WB assay. Immunoprecipitation (IP) and co‐immunoprecipitation (CO‐IP) were used for hunting the interacted proteins of MFG‐E8.

**Results:**

In vivo, sarcopenia, mitophagy deficiency, and up‐regulated MFG‐E8 were confirmed in the STZ‐P8 group. DP exerted protective effects on sarcopenia and mitophagy (DP + STZ‐P8 vs. STZ‐P8; all *P* < 0.01), such as increased lean mass (8.47 ± 0.81 g vs. 7.08 ± 1.64 g), grip strength (208.62 ± 39.45 g vs. 160.87 ± 26.95 g), rotarod tests (109.7 ± 11.81 s vs. 59.3 ± 20.97 s), muscle cross‐sectional area (CSA) (1912.17 ± 535.61 μm^2^ vs. 1557.19 ± 588.38 μm^2^), autophagosomes (0.07 ± 0.02 per μm^2^ vs. 0.02 ± 0.01 per μm^2^), and cytolysosome (0.07 ± 0.03 per μm^2^ vs. 0.03 ± 0.01 per μm^2^). DP down‐regulated MFG‐E8 in both serum (DP + STZ‐P8: 253.19 ± 34.75 pg/mL vs. STZ‐P8: 404.69 ± 78.97 pg/mL; *P* < 0.001) and gastrocnemius muscle (WB assay. DP + STZ‐P8: 0.39 ± 0.04 vs. STZ‐P8: 0.55 ± 0.08; *P* < 0.01). DP also up‐regulated PINK1, Parkin and LC3B‐II/I ratio, and down‐regulated P62 in gastrocnemius muscles (all *P* < 0.01). In vitro, mitophagy deficiency and MFG‐E8 up‐regulation were confirmed in diabetic and senescent models (all *P* < 0.05). DP and MsiRNA down‐regulated MFG‐E8 and P62, and up‐regulated PINK1, Parkin and LC3B‐II/I ratio to promote mitophagy as Torin‐1 does (all *P* < 0.05). HSPA1L was confirmed as an interacted protein of MFG‐E8 in IP and CO‐IP assay. Mover down‐regulated the expression of Parkin via the HSPA1L‐Parkin pathway, leading to mitophagy inhibition. MsiRNA up‐regulated the expression of PINK1 via SGK1, FOXO1, and STAT3 phosphorylation pathways, leading to mitophagy stimulation.

**Conclusions:**

MFG‐E8 is a crucial target protein of DP and plays a distinct role in mitophagy regulation. DP down‐regulates the expression of MFG‐E8, reduces mitophagy deficiency, and alleviates the symptoms of diabetic sarcopenia, which could be considered a novel therapeutic strategy for diabetic sarcopenia.

## Introduction

Primary sarcopenia is considered to be a progressive skeletal muscle disorder involving loss of muscle mass, strength, and function, which brings adverse outcomes such as falls, frailty, and mortality, and has been identified as age‐related skeletal muscle degeneration disease.^1^ Aside from aging, diabetes has been confirmed as a crucial risk factor that leads to sarcopenia.^2^ Characterized by muscle mass loss and physical function deterioration, sarcopenia is accompanied by poor quality of life. Diabetic sarcopenia usually manifests more progressive symptoms and brings more challenges for therapeutic strategies.

To date, the pathogenesis of sarcopenia has not been fully unravelled. Comprehensive knowledge of diabetic sarcopenia pathogenesis including key regulatory proteins, balance of the protein synthesis, and degradation systems is essential for the development of therapeutic strategies. Mitophagy, an autophagy of mitochondria, has been confirmed deficient in age‐related diseases.^3^ Recent research suggests that mitophagy deficiency may be a novel pathogenesis contributing to the development of diabetic sarcopenia.^4^

MFG‐E8, known as lactadherin, was originally identified as an epithelial cell surface protein. Aside from its positive functions including anti‐inflammation and tissue regeneration,^5^ MFG‐E8's adverse effects related to arterial aging,^6^ neuromuscular junctions (NMJs) degeneration,^7^ and melanoma tumour progression^8^ have also been reported. Our previous study reported that serum MFG‐E8 in elders with type 2 diabetes mellitus was higher than that of younger or elders without diabetes.^9^ In comparison with the positive functions of MFG‐E8, it is important to consider its adverse effects. Thus, it is reasonable to hypothesize that high levels of MFG‐E8 may be associated with diabetic sarcopenia.

D‐pinitol (DP), isolated from soybean seeds, is a natural compound that exerts versatile biological effects, such as hypoglycaemic, antioxidant, anti‐inflammatory, hepatoprotective, cardioprotective, and neuroprotective activities.^10^ Our previous studies^9^, ^11^ have elaborated on the therapeutic effects of DP on diabetic mice including hypoglycaemic and MFG‐E8 down‐regulation; however, there are no reports on DP's effect on sarcopenia. MFG‐E8 might be a key protein regulated by DP, playing an essential role in regulating mitophagy. It is involved in the pathological process of diabetic sarcopenia, which sheds light on a new therapeutic target for diabetic sarcopenia. To further investigate the therapeutic effect of DP on diabetic sarcopenia, especially the underlying mechanisms of MFG‐E8 and its regulatory effect on mitophagy, we used streptozotocin (STZ) to induce diabetes in senescence‐accelerated mouse prone 8 (SAM‐P8) and senescence‐accelerated mouse resistant 1 (SAM‐R1) in vivo and established the diabetic or senescent model with myoblast cell line in vitro.

## Methods

### Animals

SAM‐P8 (P8) and SAM‐R1 (R1) were purchased from the Department of Laboratory Animal Science of Peking University Health Science Center (Permit Number: SCXK 2016‐0010). Twelve‐week‐old healthy male SPF P8 and R1 (WT. 20–30 g) were housed in individual cages under controlled temperature (20 to 22°C) and relative humidity (40–60%), with a 12‐h light–dark cycle, and were allowed free access to distilled water and standard chow.

### Grouping and experimental procedure

After two weeks of adaptive feeding, mice were divided into five groups randomly: (A) R1 (*n* = 10), (B) STZ‐R1 (*n* = 12), (C) P8 (n = 10), (D) STZ‐P8 (n = 12), (E) DP + STZ‐P8 (n = 12). A dosage of STZ (Sigma‐Aldrich, Merck, Darmstadt, Germany) at 50 mg/kg intraperitoneal injection per day for 5 consecutive days was used to induce diabetic models. One week later, the DP + STZ‐P8 group received DP (Sigma‐Aldrich, Merck, Darmstadt, Germany. 30 mg/mL) diluted with distilled water in a dosage of 150 mg/kg per day intragastrically and the other groups received a placebo. Body weight and blood glucose (Fasting Blood Sugar, FBS) were measured every other week. Six weeks later, 51 mice completed the experiment, and body mass, whole body composition, grip strength, and rotarod tests were performed before being euthanized. The other 5 mice dropped out of the experiment when they lost more than 40% of their body weight during the week of STZ injection and were euthanized by heart bleeding. Under anaesthesia, blood was collected before the systemic circulation perfusion with normal saline. The gastrocnemius samples were harvested. The concentration of MFG‐E8 in the serum was detected by Elisa kit (Camilo Biological Co., Ltd., Nanjing, China).

### Grip strength and rotarod tests

The forelimb grip strength of mice was measured with a force gauge (Yiyan Technology Development Co., Ltd., Jinan, China). Hold the mouse tail with its fore paws grasping the grid connected to the force gauge. Then, pull slowly until the mouse releases its fore paws from the grid,^12^ and the grip strength value is recorded. After three repeated tests, the average value was recorded. An accelerating rotarod device (Yiyan Technology Development Co., Ltd., Jinan, China) was used to measure the coordination and balance of the mice.^13^ A 3‐day training was performed before the rotarod test. The acceleration settings were 40 rpm, and the average value of latency to fall from the rotating rod during three testing periods was calculated for each mouse.

### Whole body composition measurement

Whole body composition measurement was performed using dual‐energy X‐ray absorptiometry (DXA) (XR‐800, Norland, Mahwah, USA). Under general anaesthesia, mice were placed on the DXA device in a prone position with four limbs fixed for scanning. Lean mass and fat mass were measured by default in small animal software for further analysis.^14^

### Optical microscopy

The gastrocnemius samples sectioned at 5 μm thick (RM2245, Leica, Heidelberger, Germany) were subjected to haematoxylin/eosin (H&E), IHC, or IF staining.^15^ For IHC staining, slides were subjected to antigen retrieval procedure and blocked with 5% Bovine Serum Albumin (BSA), then incubated with anti‐MFG‐E8 (1:100. A12322, Abclonal, Wuhan, China), anti‐LC3B (1:200. 18,725–1‐AP, Proteintech, Wuhan, China), anti‐P62 (1:200. BA2849, Boster, Wuhan, China), or anti‐PINK1(1:200. 23,274–1‐AP, Proteintech) overnight at 4°C. Slides were incubated with HRP conjugated goat anti‐rabbit (1:200. GB23303, Servicebio, Wuhan, China) for 50 min at room temperature. DAB (3,3′‐diaminobenzidine) chromogenic reaction was performed and haematoxylin for the nuclei staining. Images of H&E and IHC were acquired with a fluorescence microscope (Olympus BX43).

For Dystrophin IF staining, slices were subjected to antigen retrieval and blocking procedures, then incubated with anti‐Dystrophin (1:100. A4746, Abclonal) at 4°C overnight, then incubated with CY3 labelled goat anti‐rabbit IgG (1:300. GB21303, Servicebio) for 1 h at room temperature. For double IF staining, the tyramide signal amplification (TSA) method was used to mark different targeting proteins. Slices were subjected to antigen retrieval and blocking procedures. Then incubation with anti‐MFG‐E8 (1:100. Abclonal) overnight at 4°C, and then incubated with HRP conjugated goat anti‐rabbit antibody (1:200. GB23303, Servicebio) for 50 min at room temperature. Subsequently, slides were incubated with TSA‐CY3 (G1223, Servicebio) solution in a dark condition for 10 min. After microwave treatment, slides were incubated with an anti‐Parkin (1:100. 14060‐1‐AP, Proteintech) overnight at 4°C, then incubated with Alexa fluor 488‐conjugated goat anti‐rabbit antibody (1:400. GB25303, Servicebio) in dark condition. Finally, the nuclei in all slices were stained by 4′,6‐diamidino‐2‐phenylindole (DAPI) (G1012, Servicebio). Images were acquired with a fluorescence microscope (Nikon Eclipse C1, Japan). Measurement of CSA was performed by ImageJ software, and at least 500 individual myofibers were measured per muscle sample.

### Transmission electron microscopy

Transmission electron microscopy was used to detect sarcomere, mitochondria, phagophore, autophagosome, and cytolysosome in gastrocnemius. The biopsy specimens were cut into cubes of 1 mm^3^ in size, fixed in glutaraldehyde solution, and then embedded in resin blocks. Specimens were cut into 70 nm‐thick ultrathin sections by ultra‐microtome (Leica EM UC7), then stained by uranium acetate saturated alcohol and lead citrate solution. The ultrathin sections were then examined by a transmission electron microscope (HITACHI HT7700, Japan) at 120 kV at the electron microscopy unit (Servicebio Biotechnology Co., LTD, Wuhan, China).

### Cell culture

A myoblast cell line (C2C12) was obtained from iCellbioscience (Shanghai, China). Cells were cultured in Dulbecco's Modified Eagle Medium (DMEM) supplemented with 10% fetal bovine serum (FBS), 100 U/mL penicillin, and 100 μg/mL streptomycin, and incubated at 37°C in 5% CO_2_/95% air. The culture medium was replaced every other day. CCK‐8 assay was used to measure cell viability of AGEs (Bioss Biotechnology Co., LTD, Beijing, China), DG (Biosharp Life Sciences Co., Ltd, Hefei, China), DP, and pre‐incubation protocols (Figure S1a,c,d). Concentrations corresponding to 60% of cell viability (100 mg/L AGEs or 30 g/L DG) for 48 h were used for the senescent or diabetic cell model.^16^ 160 μM DP was used as a protective concentration.^17^ 250 nM Torin‐1 (MCE, New Jersey, USA)^18^ or 5 mM 3‐MA (MCE)^19^ for 12 h was used as a mitophagy agonist or inhibitor. When cells were subjected to Part 1–3 protocols (Figure S1b), FBS was changed to 2% horse serum for differentiation.

### Transfection of plasmid DNA and siRNA

The plasmid Mus‐MFG‐E8 (NM_008594.2)‐C‐FLAG (PCDNA3.1, Boshang Biotechnology Co., LTD, Shanghai, China) and the siRNA MFG‐E8‐Mus‐1,029 (GenePharma Co., Ltd, Shanghai, China) were purchased. Lipofectamine 3,000 (ThermoFisher, LTU) was used for the transfection of plasmid or siRNA; 3 μg/mL of plasmid or 2.5 μL/mL of siRNA was used in the transfection assay. Transfection efficiency was evaluated by green fluorescence emitted by GFP (plasmid) or FAM (siRNA) with a fluorescence microscope (CKX53, Olympus), real‐time fluorescent quantitative PCR (qPCR), and WB assay (Figure S2a–c). The primers were purchased from KeyGEN BioTECH Co., Ltd. (Nanjing, China). Alpha‐actin, AAGTCCTGCAAGTGAACAAGC, and AGGTGTGGTGCCAGATCTTC; MFG‐E8, CACTGTGAAACCGGTTGTTCT, and CTCCTTGTCTCCACCGCTTT.

### Mitophagy staining

Mitophagy staining was performed to detect the level of mitophagy according to the manufacturer's instructions. 10^4^ cells per well were seeded in a 12‐well plate and were incubated overnight. Then the mitophagy dye (DOJINDO chemical technology, Co., Ltd, Shanghai, Japan) was added to wells and then incubated for about half an hour. Then cells were subjected to Part 1–3 protocols (Figure S1b). Images were acquired with a fluorescence microscope (Olympus, CKX53, Japan) and the mitophagy area was evaluated by ImageJ software.

### Western blotting assay

Radioimmunoprecipitation (RIPA) lysis buffer (SW104, Seven biotech, Beijing, China) with 1 mM phenylmethanesulfonyl fluoride (PMSF) (SW106, Seven) and protein phosphatase inhibitor (P1260, Solarbio, Beijing, China) was used for the protein extracts from whole cells and gastrocnemius specimens. SDS‐page gels (8–15%) were used to separate proteins, and the separated proteins were transferred to the polyvinylidene fluoride (PVDF) membrane (Millipore, Merck, Darmstadt, Germany). After being blocked with nonfat milk (5%) for 90 min at room temperature, the membrane was incubated with primary antibodies overnight at 4°C and then incubated with secondary antibodies for 60 min at room temperature. Protein band visualization was performed using the Immobilon ECL Ultra Western HRP Substrate Kit (Millipore) and automatic chemiluminescence image analysis system (Tanon4800, USA).

Primary antibodies: anti‐MFG‐E8 (1 μg/mL. AF2805, R&D SYSTEMS, Minnesota, USA), anti‐LC3B (1:1000), anti‐P62 (1:1000), anti‐PINK1 (1:1000), anti‐Parkin (1:1000), anti‐α‐actin (1:1000. 23,660–1‐AP, Proteintech), anti‐GAPDH (1:5000. 10,494–1‐AP, Proteintech), anti‐P16 (1:1000. A0262, Abclonal), anti‐P21 (1:1000. BM4382, Boster), anti‐FOXO1 (1:1000. A2934, Abclonal), anti‐p‐FOXO1 (1:1000. AF3417, Affinity, Nanjing, China), anti‐SGK1 (1:1000. A3936, Abclonal), anti‐p‐SGK1 (1:500. AF3001, Affinity), anti‐STAT3 (1:500. 10,253–2‐AP, Proteintech), anti‐p‐STAT3 (1:1000. AP0715, Abclonal), and anti‐HSPA1L (1:1000. 13,970–1‐AP, Proteintech). Second antibodies: HRP rabbit anti‐goat IgG(H + L) (1:5000, AS029, Abclonal) and HRP goat anti‐rabbit IgG (H + L) (1:5000, SA00001–2, Proteintech).

### IP and CO‐IP assay

Cell lysates (NC and Mover) were extracted in a non‐denaturing method according to the IP/CO‐IP extraction kit's (Abbkine) instructions. The lysates were incubated with beads conjugated by rabbit anti‐MFG‐E8 (Abbexa, Cambridge, UK) or rabbit IgG at 4°C overnight. Then the beads were washed, and 20 μL beads of anti‐MFG‐E8 were sent for LC–MS/MS analysis, other beads were boiled in an SDS loading buffer for WB assay, and the second antibody was HRP conjugated mouse anti‐rabbit IgG LCS (1:1000. Abbkine Scientific Co., Ltd, Wuhan, China). The mass spectrometry database retrieval software used in LC–MS/MS was MaxQuant 1.6.17.0. The protein database was sourced from UniProt‐ *Mus musculus* (Mouse) [10090]‐88079‐20220208.FASTA.

### Cell immunofluorescence and immunofluorescent co‐localization staining

Glass coverslips were placed in a 12‐well plate followed by 10^4^ cells seeded per well, and cells were subjected to Part 1–3 protocols (Figure S1b). Subsequently, cells were fixed with 4% Paraformaldehyde (PFA) (Beyotime Biotechnology, Shanghai, China) for 15 min, and then the membrane was penetrated by 0.5% Triton X‐100 (Solarbio), and antigens were blocked with 5% BSA. Then coverslips were incubated with primary antibodies (anti‐MFG‐E8 or anti‐Parkin; a cocktail of rabbit anti‐MFG‐E8 and mouse anti‐HSPA1L for immunofluorescent co‐localization assay) at 4°C overnight. Subsequently, the coverslips were incubated with fluorescence staining secondary antibodies for 60 min at room temperature. MFG‐E8 with Alexa Fluor 594 (red), and Parkin with Alexa Fluor 488 (green) were for IF assay respectively. The cocktail of Alexa Fluor 594 (MFG‐E8) and 488 (HSPA1L) was for co‐localization assay. The nuclei were stained with DAPI. Images were obtained by a fluorescence microscope (Olympus, BX43, Japan) and evaluated by ImageJ software. Primary antibodies: anti‐MFG‐E8 (1:100. Abclonal), anti‐Parkin (1:50) and anti‐HSPA1L (1:100. 66780‐1‐Ig, Proteintech). Second antibodies: CoraLite594 goat anti‐rabbit (1:400. SA00013‐4, Proteintech), CoraLite488 goat anti‐rabbit (1:100. SA00013‐2, Proteintech), and CoraLite488 goat anti‐mouse (1:100. SA00013‐1, Proteintech).

### Statistical analysis

Where applicable, all data were presented as means ± standard deviation. Comparison of data between groups was performed using a one‐way analysis of variance (ANOVA), and the LSD method was used for further multiple comparisons when there were differences between groups via the one‐way ANOVA analysis. All statistical analyses were performed using the Statistic Package for Social Science (SPSS 22.0). Probability values <0.05 were considered to be statistically significant.

## Results

### DP exerts protective effects on the diabetic sarcopenia model

**Figure 1:**
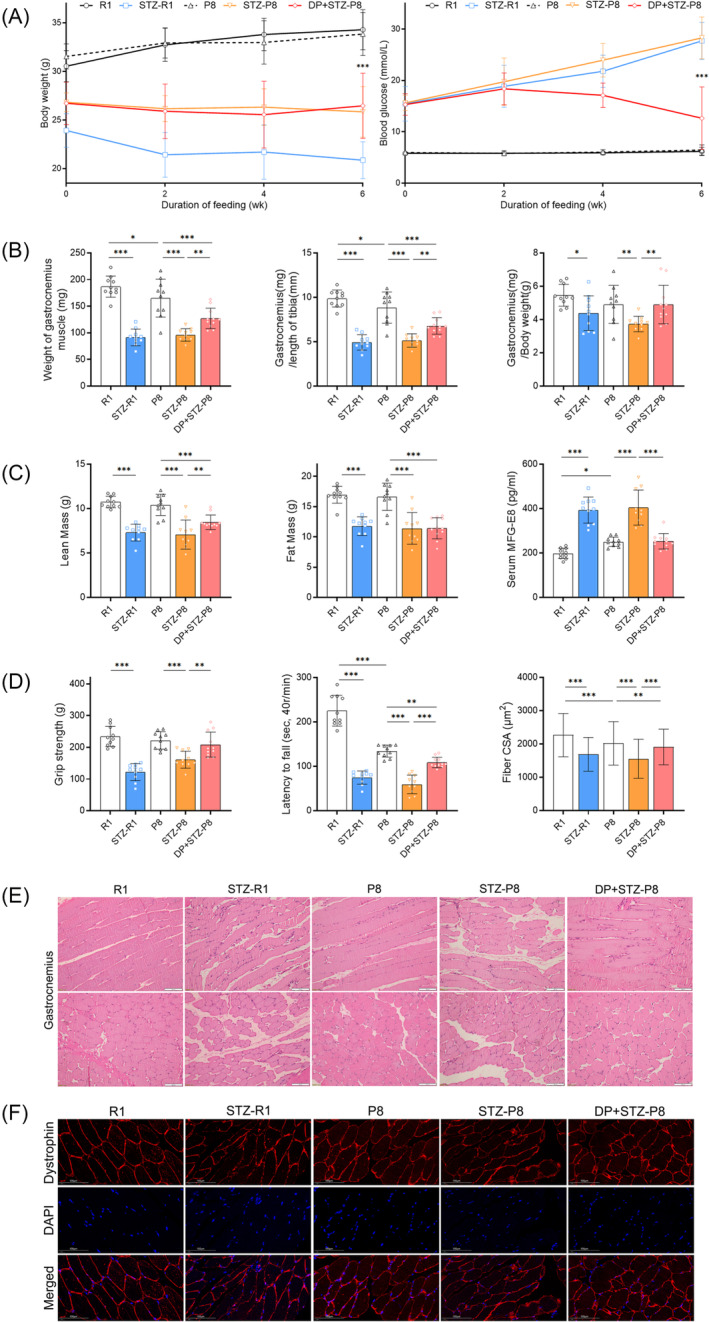
Body (or gastrocnemius) weight, glucose, body composition, grip strength, rotarod, and histological results. (A) Weight and blood glucose of raw data. Body weight and glucose of 0 week, 2 weeks, 4 weeks, 6 weeks: The difference between STZ‐R1 and R1 was statistically significant (*P* < 0.001), and so were STZ‐P8 (or DP + STZ‐P8) and P8 (all *P* < 0.001). The difference between DP + STZ‐P8 and STZ‐P8 was statistically significant in 4w and 6w in glucose (*P* < 0.001). (B) Weight of gastrocnemius. The difference between STZ‐R1 (or P8) and R1 was statistically significant (all *P* < 0.05), and so were P8 and STZ‐P8 (*P* < 0.01). The difference between DP + STZ‐P8 (127.18 ± 19.18 mg) and STZ‐P8 (96 ± 11.74 mg) was statistically significant (*P* < 0.01). The ratio of gastrocnemius to body weight and gastrocnemius to the length of the tibia has a similar trend to the weight of gastrocnemius. (C) Body composition analysis and serum MFG‐E8. Lean mass: The difference between STZ‐R1 and R1 was statistically significant (*P* < 0.001), and so were STZ‐P8 (or DP + STZ‐P8) and P8 (all *P* < 0.001). The difference between DP + STZ‐P8 (8.47 ± 0.81 g) and STZ‐P8 (7.08 ± 1.64 g) was statistically significant (*P* < 0.01). Fat mass: The difference between STZ‐R1 and R1 was statistically significant (*P* < 0.001), and so were STZ‐P8 (or DP + STZ‐P8) and P8 (all *P* < 0.001). The serum MFG‐E8: The difference between STZ‐R1 (or P8) and R1 was statistically significant (all *P* < 0.05), and so were STZ‐P8 and P8 (*P* < 0.001). The difference between DP + STZ‐P8 (253.19 ± 34.75 pg/mL) and STZ‐P8 (404.69 ± 78.97 pg/mL) was statistically significant (*P* < 0.001). (D) Grip strength, rotarod test and fibre CSA. Grip strength: The difference between STZ‐R1 and R1 was statistically significant (*P* < 0.001), and so was STZ‐P8 and P8 (*P* < 0.001). The difference between DP + STZ‐P8 (208.62 ± 39.45 g) and STZ‐P8 (160.87 ± 26.95 g) was statistically significant (*P* < 0.01). Rotarod test: The difference between STZ‐R1 (or P8) and R1 was statistically significant (all *P* < 0.001), and so were STZ‐P8 (or DP + STZ‐P8) and P8 (all *P* < 0.01). The difference between DP + STZ‐P8 (109.7 ± 11.81 s) and STZ‐P8 (59.3 ± 20.97 s) was statistically significant (*P* < 0.001). Fibre CSA: The difference between STZ‐R1 (or P8) and R1 was statistically significant (all *P* < 0.001), and so were STZ‐P8 (or DP + STZ‐P8) and P8 (all *P* < 0.01). The difference between DP + STZ‐P8 (1912.17 ± 535.61 μm^2^) and STZ‐P8 (1557.19 ± 588.38 μm^2^) was statistically significant (*P* < 0.001). (E) Histological images of gastrocnemius with H&E staining. Scale bar = 100 μm. (F) Images of dystrophin immunofluorescence (red) in gastrocnemius muscle cross‐sections. Nuclei were counterstained with DAPI (blue). Scale bar = 100 μm. D‐pinitol (DP), cross‐sectional area (CSA) (*P < 0.05; **P < 0.01; ***P < 0.001).

The body weight, weight of gastrocnemius muscle, the ratio of gastrocnemius/length of the tibia (or body weight), lean mass, grip strength, and the time of latency to fall were significantly decreased in STZ‐P8 (or STZ‐R1) group in comparison with P8 (or R1) group (all *P* < 0.05); besides, the blood glucose was significantly increased in STZ‐P8 (or STZ‐R1) group when compared with P8 (or R1) group (all *P* < 0.001) (Figure 1A–D). The body weight changes in the 2 weeks, 4 weeks, and 6 weeks were detailed in Figure S3a. The weight of the gastrocnemius muscle and the time of latency to fall were significantly decreased in the P8 group in comparison with the R1 group (all *P* < 0.05) (Figure 1B,C). The pathological features of sarcopenia including atrophy, a disorder of fibres, and CSA reduction were inconspicuous in 22‐week‐old P8 mice, while these features were conspicuous in STZ‐P8 and STZ‐R1 groups (Figure 1E,F). After 6 weeks of DP treatment, the blood glucose in the DP + STZ‐P8 group (12.65 ± 6.11 mmol/L) was significantly decreased in comparison with the STZ‐P8 group (28.3 ± 4.06 mmol/L) (*P* < 0.001). There is no statistical difference in body weight between DP + STZ‐P8 and STZ‐P8 groups (Figure 1A). In the DP + STZ‐P8 group, the weight of gastrocnemius muscle (127.18 ± 19.18 mg vs. 96 ± 11.74 mg), the ratio of gastrocnemius/length of the tibia (or body weight), the lean mass (8.47 ± 0.81 g vs. 7.08 ± 1.64 g), grip strength (208.62 ± 39.45 g vs. 160.87 ± 26.95 g), and the time of latency to fall (109.7 ± 11.81 s vs. 59.3 ± 20.97 s) were significantly improved when compared with STZ‐P8 group (all *P* < 0.01) (Figure 1B–D). Besides, the pathological features of sarcopenia alleviated significantly (Figure 1E), and fibre CSA improved significantly (1912.17 ± 535.61 μm^2^ vs. 1557.19 ± 588.38 μm^2^, *P* < 0.001) in comparison with STZ‐P8 group (Figure 1D,F).

### Mitophagy deficiency in diabetic sarcopenia model and MFG‐E8 participated in mitophagy regulation

**Figure 2:**
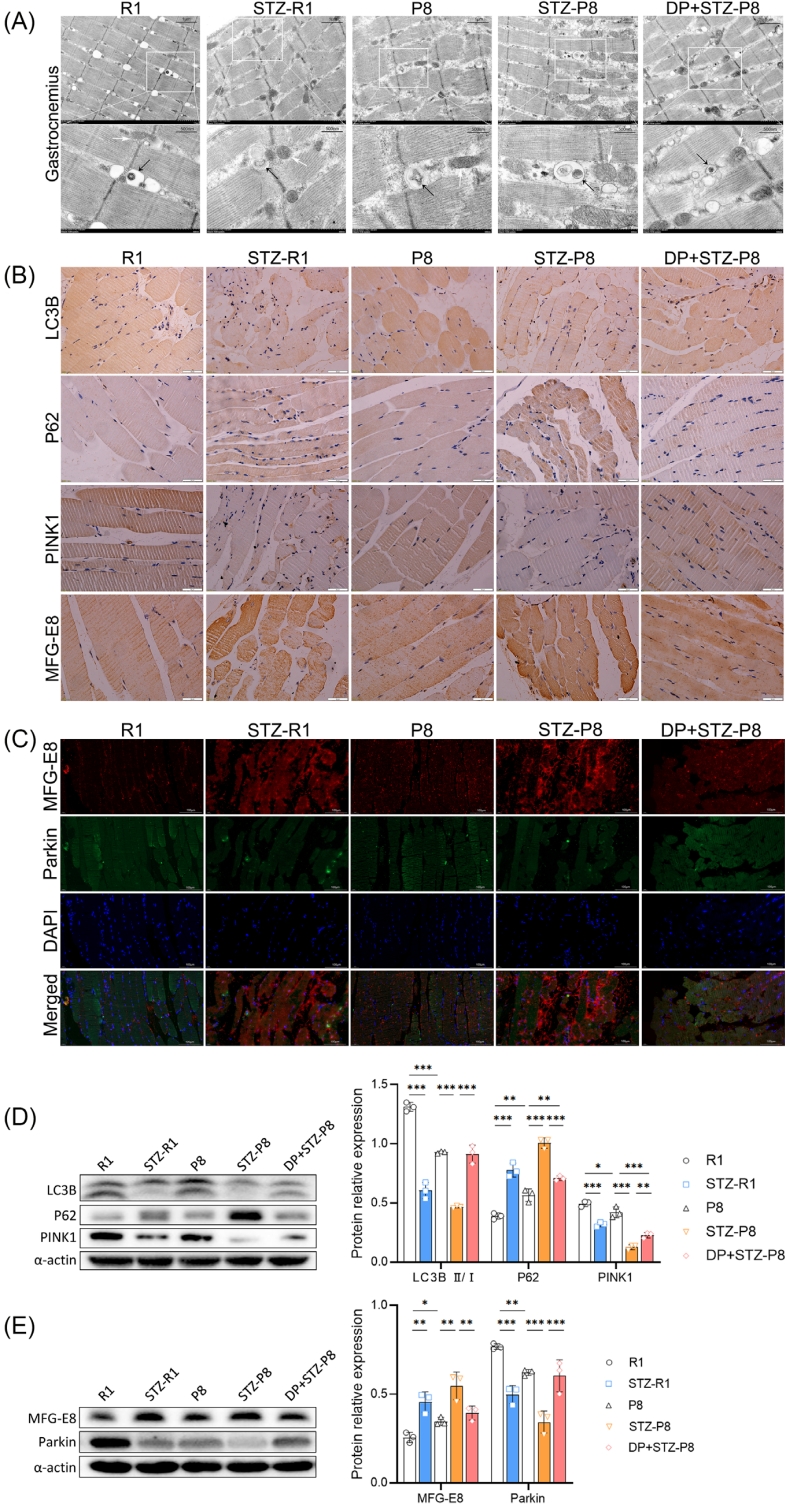
Images of electron microscopy, immunohistochemistry (IHC) and immunofluorescence (IF), WB strips of LC3B, P62, PINK1, MFGE‐8, and Parkin, and quantitative analysis in vivo. (A) Transmission electron microscope images of the gastrocnemius. The black arrow represents autophagosome or autolysosome with mitochondria packaged inside. The white arrow represents mitochondria. Scale bar = 1 μm or 500 nm. (B) IHC images of the gastrocnemius. Scale bar = 50 μm. (C) IF staining images of the gastrocnemius. Scale bar = 100 μm. (D) WB strips of LC3B, P62, PINK1, and quantitative analysis. (E) WB strips of MFG‐E8, Parkin, and quantitative analysis (*P < 0.05; **P < 0.01; ***P < 0.001).

Mitophagy deficiency including mitochondrial ridge disorder, damaged mitochondria accumulation, autophagosome reduction, and cytolysosome deficiency was confirmed in P8, STZ‐R1, and STZ‐P8 groups (Figure 2A). After DP treatment, the damaged mitochondria decreased significantly (DP + STZ‐P8 vs. STZ‐P8) (Figure 2A), and autophagosome (0.07 ± 0.02 per μm^2^ vs. 0.02 ± 0.01 per μm^2^, *P* < 0.001) and cytolysosome (0.07 ± 0.03 per μm^2^ vs. 0.03 ± 0.01 per μm^2^, *P* < 0.01) increased significantly in comparison with STZ‐P8 group (Figure S3b). Serum MFG‐E8 was up‐regulated in STZ‐R1, P8 and STZ‐P8 groups when compared with the R1 (or P8) group (all *P* < 0.05) (Figure 1C). In the STZ‐R1, P8 and STZ‐P8 groups, MFG‐E8 was up‐regulated and Parkin was significantly down‐regulated, which can be identified in IHC images (Figure 2B), IF images (Figure 2C), and WB assay when compared with the R1 (or P8) group (all *P* < 0.05) (Figure 2E); PINK1 and LC3B (or II/I ratio) in these groups were down‐regulated, and P62 was up‐regulated, which can be identified in IHC images (Figure 2B) and WB assay when compared with the R1 (or P8) group (all *P* < 0.05) (Figure 2D). After DP intervention, Parkin, LC3B (or II/I ratio), and PINK1 were up‐regulated, and MFG‐E8 and P62 were down‐regulated significantly when compared with STZ‐P8 group, which can be identified in IHC and IF images (Figure 2B,C), and WB assay (all *P* < 0.01) (Figure 2D,E).

### DP and MsiRNA's anti‐senescent effects in vitro

The cell viability of AGEs or DG presented a distinct dose‐dependent manner, while DP presented a non‐dose‐dependent manner (Figure S1c). The pre‐incubation protocol manifested significantly increased cell viability than the co‐incubation protocol (Figure S1d) (all *P* < 0.05). For the sake of brevity, we named these pre‐incubation protocols as DP + AGEs and DP + DG.

The SA‐β‐gal staining results showed that AGEs, DG, and Mover caused heavy dyeing, while the DP and MsiRNA alleviated the staining significantly (Figure S4a). WB results showed that P21 and P16 were up‐regulated in AGEs, DG, and Mover groups when compared with the CC (or GFP) group (all *P* < 0.01) (Figure S4b,c). P21 and P16 were down‐regulated after the intervention of DP or MsiRNA in comparison with pre‐intervention (all *P* < 0.01) (Figure S4b,c). It indicated that DP and MFG‐E8 downregulation inhibited processes associated with cell senescence.

### Effects of MFG‐E8 (or DP) on mitophagy, LC3B‐II/I ratio, P62 and PINK1 in vitro

**Figure 3:**
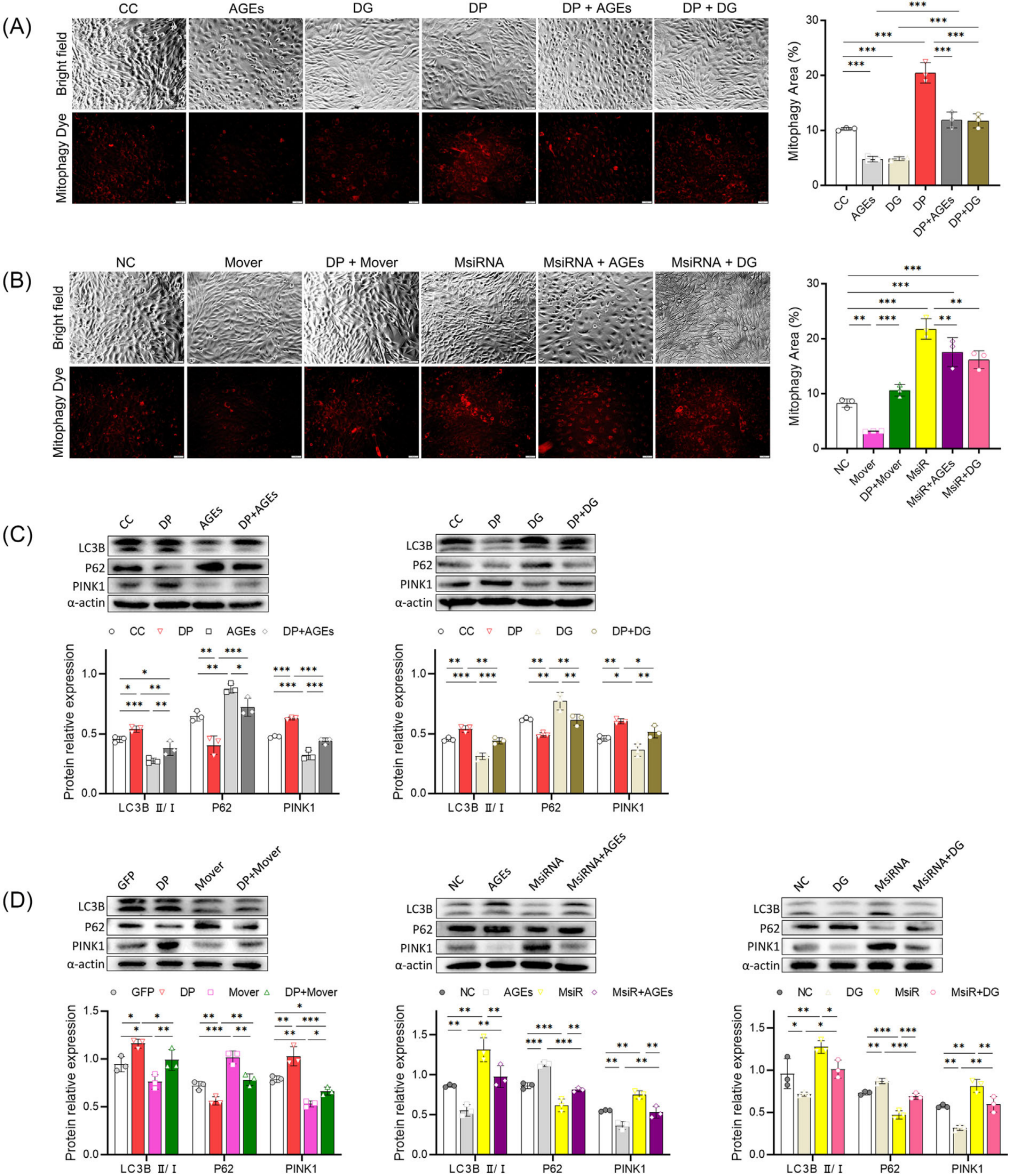
Images of mitophagy staining, WB strips of LC3B, P62, and PINK1, and quantitative analysis in vitro. (A) Images of mitophagy staining in part 1 protocol. Scale bar = 50 μm. Mitophagy area: The difference between AGEs (DG or DP) and CC was statistically significant (all *P* < 0.001). The difference between DP + AGEs (11.92 ± 1.44%) (or DG, 11.76 ± 1.29%) and AGEs (or DG) was statistically significant (all *P* < 0.001). (B) Images of mitophagy staining in part 3 protocol. Scale bar = 50 μm. Mitophagy area: The difference between Mover (or MsiRNA) and NC was statistically significant (all *P* < 0.01). The difference between DP + Mover (10.63 ± 1.05%) and Mover, and the difference between MsiRNA+AGEs (17.58 ± 2.63%) (or DG, 16.21 ± 1.64%) and MsiRNA was statistically significant (all *P* < 0.01). (C) WB strips in part 1 protocol and quantitative analysis. (D) WB strips in part 3 protocol and quantitative analysis. Abbreviations: D‐galactose (DG), advanced glycosylation end products (AGEs), MFG‐E8 overexpression (Mover), MFG‐E8 siRNA (MsiRNA) (*P < 0.05; **P < 0.01; ***P < 0.001).

The red fluorescence and its area reflect the level of mitophagy, which was weakened (decreased) in the AGEs, DG, Mover, and 3‐MA group in comparison with CC (or NC) (all *P* < 0.01) (Figure 3A,B; Figure S5a). After the intervention of DP (DP + AGEs, DP + DG, DP + Mover, and DP + 3‐MA groups), the red fluorescence enhanced, and the mitophagy area increased significantly in comparison with pre‐intervention (all *P* < 0.001) (Figure 3A,B; Figure S5a). Like Torin‐1 and DP, MsiRNA enhanced the red fluorescence and its areas and alleviated the mitophagy inhibition caused by AGEs (or DG) in MsiRNA+AGEs/DG groups (Figure 3A,B; Figure S5a). WB results manifested that LC3B‐II/I ratio and PINK1 were down‐regulated, and P62 was up‐regulated in AGEs, DG, Mover, and 3‐MA groups in comparison with CC (or GFP) (all *P* < 0.05) (Figure 3C,D; Figure S5b). After DP intervention (DP + AGEs, DP + DG, DP + Mover, and DP + 3‐MA groups), LC3B‐II/I ratio and PINK1 were up‐regulated, and P62 was down‐regulated in comparison with pre‐intervention (all *P* < 0.05) (Figure 3C,D; Figure S5b). Like Torin‐1 and DP, MsiRNA up‐regulated LC3B‐II/I ratio and PINK1, and down‐regulated P62 significantly in comparison with CC (or NC) (all *P* < 0.05) (Figure 3C,D). MsiRNA also reverses the trend caused by AGEs (or DG) in MsiRNA+AGEs/DG groups (all *P* < 0.05), thereby alleviating the mitophagy inhibition.

### Effects of MFG‐E8 on the mitochondrial membrane potential (MMP) in vitro

From the images of the JC‐1 staining, MMP inhibition including weakened red fluorescence and enhanced green fluorescence, was confirmed in AGEs, DG, Mover, and 3‐MA groups (Figure S6). The ratio of red/green fluorescence intensity evaluated by the flow cytometry (FCM) was decreased in AGEs, DG, Mover, and 3‐MA groups in comparison with CC (or NC) (all *P* < 0.01) (Figure S7). After the intervention of DP (DP + AGEs, DP + DG, or DP + 3‐MA group), MMP inhibition was alleviated in the images, and the red/green fluorescence intensity was increased in comparison with pre‐intervention (all, *P* < 0.05) (Figures S6 and S7). Like DP, Torin‐1 alleviated the MMP inhibition in the images and increased the red/green intensity in comparison with CC (all *P* < 0.05). MsiRNA also reverses the trend caused by AGEs (or DG) in MsiRNA+AGEs/DG groups (Figures S6 and S7).

### Effects of MFG‐E8 (or DP) on the expression of Parkin in vitro

**Figure 4:**
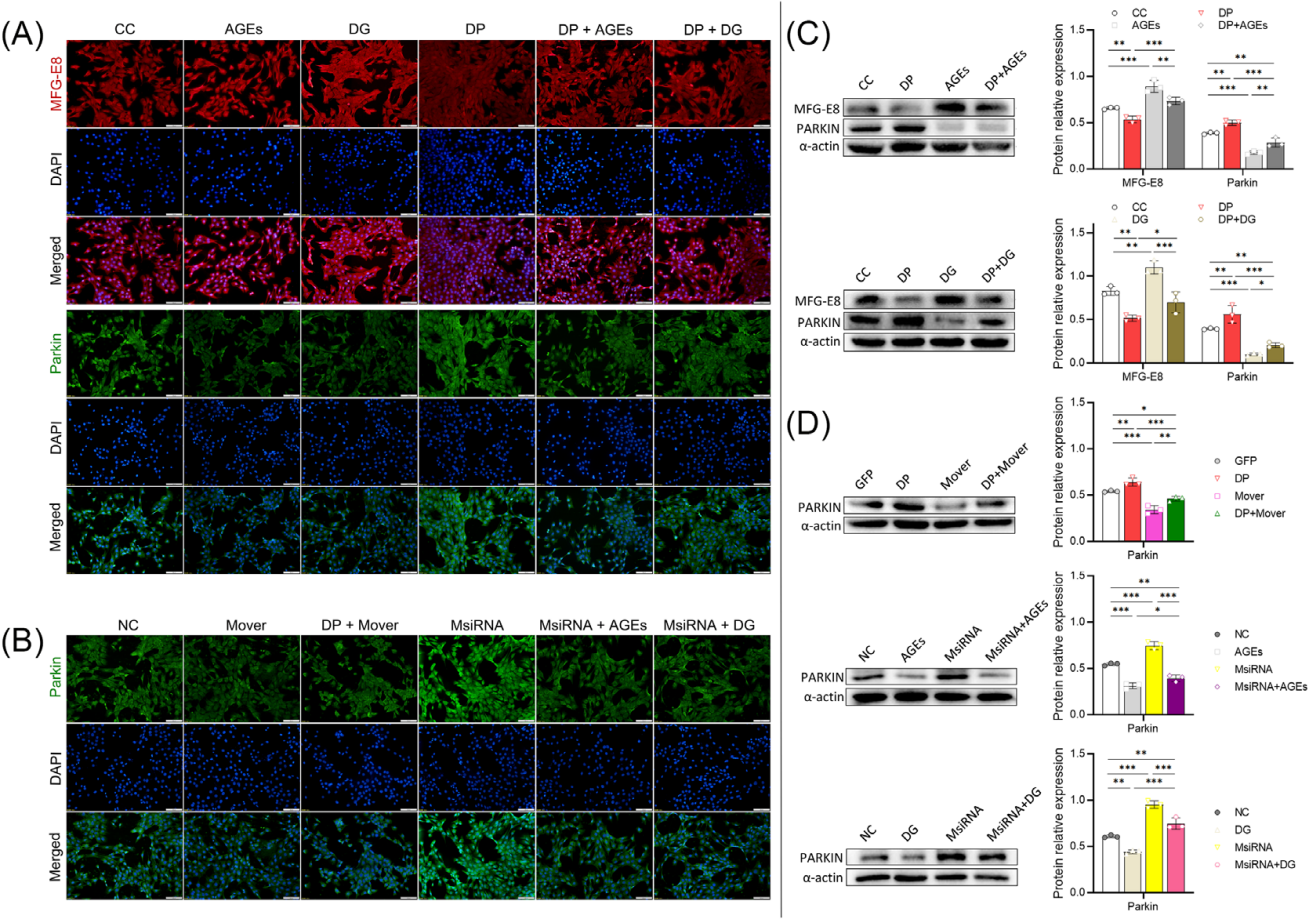
IF images of MFG‐E8 and Parkin; WB strips of MFG‐E8 and Parkin, and quantitative analysis in vitro. (A) IF images of part 1 protocol. Scale bar = 100 μm. (B) IF images of part 3 protocol. Scale bar = 100 μm. (C) WB strips in part 1 protocol and quantitative analysis. (D) WB strips in part 3 protocol and quantitative analysis (*P < 0.05; **P < 0.01; ***P < 0.001).

From the images of IF, red fluorescence (MFG‐E8) was enhanced in AGEs, DG, and 3‐MA groups, and green fluorescence (Parkin) was weakened in AGEs, DG, 3‐MA and Mover groups (Figure 4A,B; Figure S8a). After the intervention of DP, the red fluorescence was weakened in DP + AGEs, DP + DG, and DP + 3‐MA groups, the green fluorescence was enhanced in DP + AGEs, DP + DG, DP + 3‐MA, and DP + Mover groups in comparison with pre‐intervention (Figure 4A,B; Figure S8a). In the WB assay, MFG‐E8 was up‐regulated and Parkin was down‐regulated in AGEs, DG, Mover, and 3‐MA groups in comparison with CC (or GFP) (all *P* < 0.05) (Figure 4C,D; Figure S8b). After the intervention of DP (DP + AGEs, DP + DG, DP + Mover, and DP + 3‐MA groups), MFG‐E8 was down‐regulated and Parkin was up‐regulated in comparison with pre‐intervention (all *P* < 0.05) (Figure 4C,D; Figure S8b). Like DP, MsiRNA and Torin‐1 up‐regulated the expression of Parkin (all *P* < 0.01). MsiRNA reversed the trend caused by AGEs (or DG) in MsiRNA+AGEs/DG group (all *P* < 0.05), and Torin‐1 also reversed the trend caused by AGEs (or DG) in Torin‐1 + AGEs/DG group (all *P* < 0.01) (Figure 4C,D; Figure S8b).

### HSPA1L is an interacting protein of MFG‐E8 in C2C12 cells

**Figure 5:**
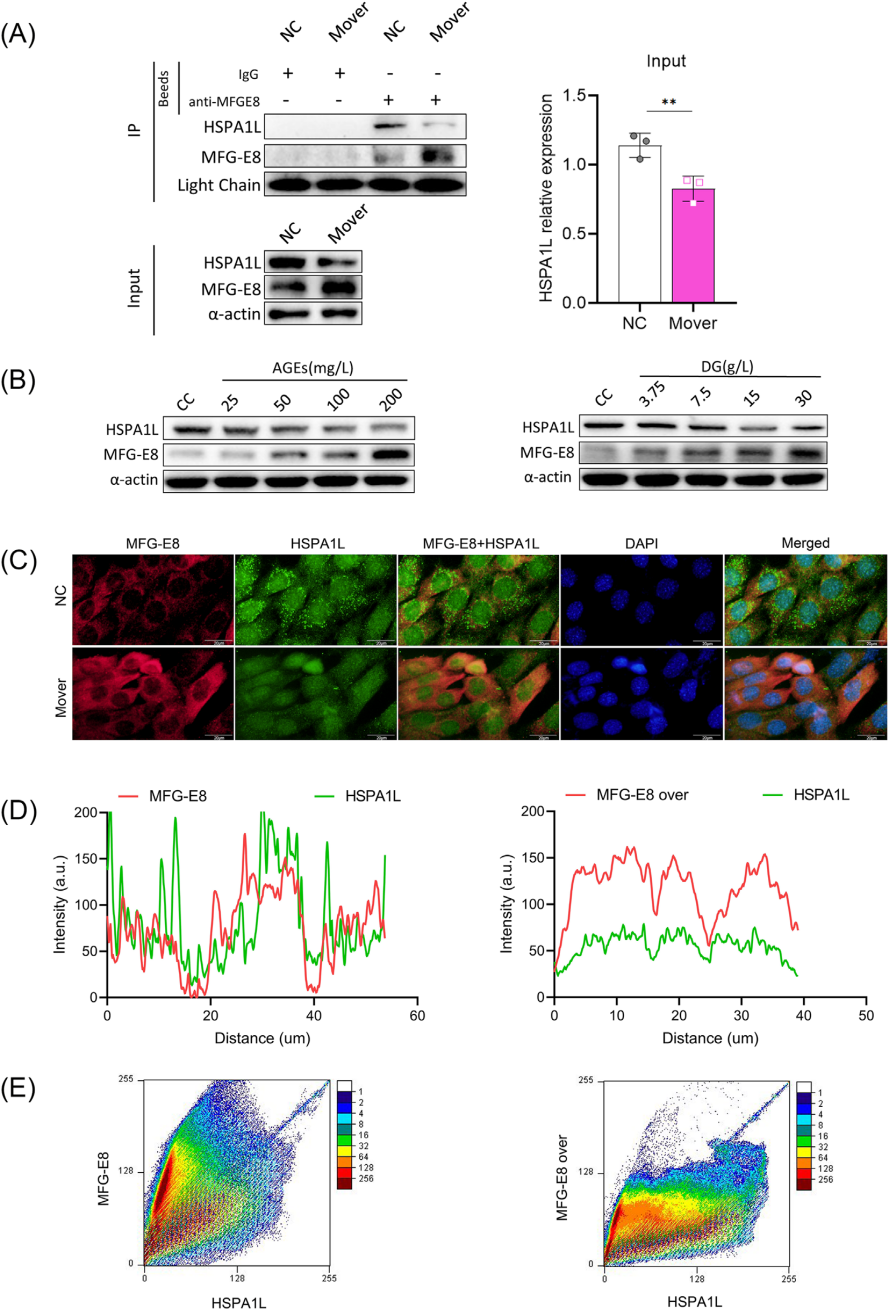
CO‐IP results and the relationship between MFG‐E8 and HSPA1L in vitro. (A) CO‐IP results and quantitative analysis of input. The HSPA1L was down‐regulated significantly when the MFG‐E8 overexpression (NC: 1.14 ± 0.09 vs. Mover: 0.83 ± 0.09, *P* < 0.01). (B) WB strips of MFG‐E8 and HSPA1L with different concentrations of AGEs (or DG) intervention. (C) Immunofluorescent co‐localization images of MFG‐E8 and HSPA1L. MFG‐E8 was stained with red fluorescence and HSPA1L was green. Scale bar = 20 μm. (D) The overlap analysis of MFG‐E8 and HSPA1L. (E) Scatter analysis of MFG‐E8 and HSPA1L (**P < 0.01).

The LC–MS/MS analysis showed that HSPA1L with a higher ranking and score was expected to be an important interacting protein of MFG‐E8 in C2C12 cells (Figure S9; Table S1). As an interacted protein of Parkin, HSPA1L is a promotor of mitophagy which has been reported previously.^20^ The CO‐IP results confirmed the presence of HSPA1L in the beads incubated with anti‐MFG‐E8, but not in the beads incubated with IgG (Figure 5A). HSPA1L was down‐regulated significantly when MFG‐E8 was over‐expressed (NC: 1.14 ± 0.09 vs. Mover: 0.83 ± 0.09, *P* < 0.01) (Figure 5A), which was confirmed again by different dosages of AGEs or DG intervention (Figure 5B). The immunofluorescent co‐localization results manifested that the consistency of HSPA1L and MFG‐E8 was significantly improved in the NC group when compared with the Mover group (NC: 0.71 ± 0.02 vs. Mover: 0.64 ± 0.04, *P* < 0.05) (Figure 5C–E).

### Role of MFG‐E8 in regulating signalling pathways related to mitophagy

**Figure 6:**
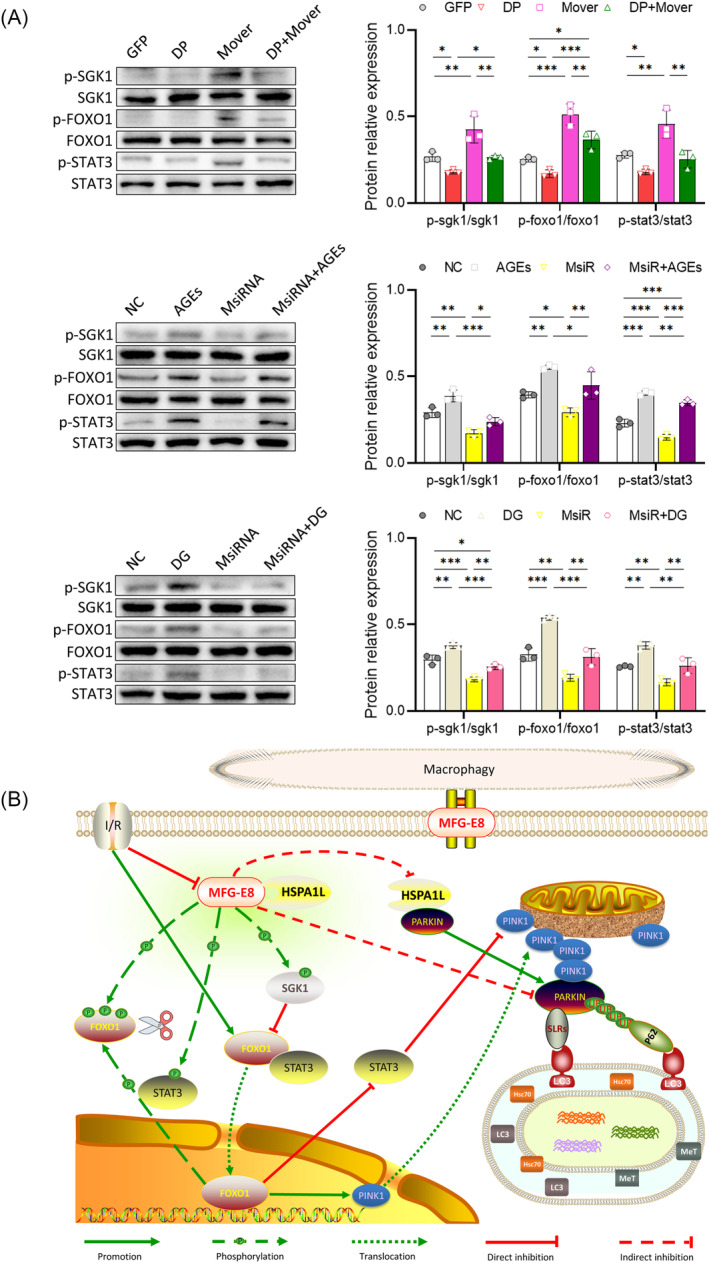
WB strips and quantitative analysis of SGK, FOXO1, and STAT3. The diagram of the signalling pathway. (A) WB strips of SGK, FOXO1, and STAT3 in part 3 protocol, and quantitative analysis (*P < 0.05; **P < 0.01; ***P < 0.001). (B) The diagram of the signalling pathway related to MFG‐E8 and mitophagy.

The Mover, AGEs, and DG up‐regulated the phosphorylation of FOXO1, SGK1, and STAT3 in WB assay in comparison with CC (or GFP) (all *P* < 0.01) (Figure 6A). DP (or MsiRNA) down‐regulated this phosphorylation caused by Mover (AGEs or DG) (all *P* < 0.05) (Figure 6A). DP exerted a positive effect on FOXO1 and PINK1 on mitophagy. MsiRNA up‐regulates the level of PINK1 via SGK1, STAT3, and FOXO1 signalling pathways, leading to mitophagy stimulation. Mover down‐regulates the level of Parkin via the HSPA1L‐Parkin pathway, thereby inhibiting mitophagy (Figure 6B).

## Discussion

Sarcopenia has become a global public health issue along with the rapidly increasing old population. It is reported that the prevalence of aging sarcopenia is 1% to 30% in a community setting.^21^ The underlying mechanisms of primary sarcopenia have not been fully revealed. However, physicians have to deal with the secondary sarcopenia caused by malnutrition,^15^ disuse, inflammation, oxidative stress, and diabetes.^22^ Mitochondria are highly dynamic organelles involved in cell metabolism maintenance. Upsetting the balance of mitochondria apoptosis including mitochondrial decline, dysfunction, and invalid quality control, deteriorates the homeostasis of skeletal muscle.^23^ Based on the results of transmission electron microscopy, it has been found that mitophagy deficiency, such as mitochondrial ridge disorder, accumulation of damaged mitochondria, reduction of autophagosome, and cytolysosome deficiency, is tightly linked to the onset of diabetic sarcopenia. Regulation of mitochondrial homeostasis might be a therapeutic strategy for diabetic sarcopenia.^4^

DP is a natural compound mainly isolated from *Ceratonia siliqua* L.(carob) and has been intensively reported recently.^24^ DP exerts diverse medicinal properties, such as anti‐Alzheimer, anti‐inflammatory, hepato‐protection, anti‐osteoporosis,^25^ anti‐aging,^26^ and anti‐tumour effect via TNF‐α and NF‐κb pathway suppression.^27^ It is considered to be a bioactive compound in diabetic mellitus therapy due to its insulin‐mimetic effect via a post‐receptor pathway of insulin action, which increases liver glucose uptake^28^ and insulin sensitivity of skeletal muscle.^29^ DP has been found to regulate oxidative stress through PI3K/Akt/mTOR pathway, which contributes to protecting the kidney from fibrosis.^30^ Additionally, it is believed to prevent or delay the onset of diabetic cardiomyopathy (DCM).^31^ Moreover, DP has been shown to affect the phosphorylated proteins related to insulin and glucagon signalling pathways, insulin resistance, and mitophagy in the heart. This, in turn, helps alleviate DCM.^32^ Our research findings suggest that the deficiency of mitophagy is reduced by DP via MFG‐E8 regulation, which contributes to alleviating symptoms of diabetic sarcopenia.

PINK1/Parkin, BINP3, Mul1, and Mdm2 are critical proteins regulating mitophagy in skeletal muscle through polyubiquitination.^33^ LC3, ULK1, and Beclin‐1 regulate the formation of autophagosomes.^34^ P62/SQSTM1 and BCL‐2/BNIP bind their cargo‐binding domain to the autophagosome membrane.^34^ Parkin's substrates including Drp1, Mfn1/2, and TOMM20, recruit the LC3, P62, and NBR1 to the surface of the mitochondria.^35^ Finally, the damaged mitochondria are encased with autophagosomes and eliminated.

FOXO1 regulates mitophagy in muscle and promotes mitophagy via the PINK1/Parkin pathway.^36^ SGK1 inhibits FOXO1, LC3 lipidation, and BECN1, leading to mitophagy inhibition.^37^ Cytoplasmic STAT3 inhibits FOXO1, and then blocks the mitophagy process. Nuclear STAT3 up‐regulates BCL2 expression, activates BCL2L1 and MCL1, then leads to mitophagy inhibition.^38^ Phosphorylated SGK1 and STAT3 facilitate the phosphorylation process of FOXO1, which inhibits mitophagy. It has been reported that the expression of P62 and LC3B‐II/I ratio varies during the aging process,^39^ however, up‐regulated P62 and down‐regulated LC3B‐II/I ratio were confirmed in this study along with the diabetic sarcopenia process.

MFG‐E8 was originally discovered in milk fat globules, and it has been identified in various cell types, including epithelial cells, vascular smooth muscle cells, dendritic cells, as well as muscle cells recently.^35^ MFG‐E8 exerts diverse biological effects. Primarily, it is considered an ‘eat‐me’ signal provider for binding apoptotic cells and macrophages^40^ and is proven to be an essential inflammatory mediator involved in cardiovascular diseases.^41^ In comparison, MFG‐E8 accumulation has been reported in arterial walls and NMJs along with aging, deteriorating cardiovascular diseases and sarcopenia.^7^, ^42^ This study confirms that MFG‐E8 is up‐regulated during diabetic sarcopenia process and it inhibits mitophagy by down‐regulating Parkin, PINK1, and LC3B‐II/I ratio.

Heat shock protein family A member 1‐like (HSPA1L) protein is an interacting protein of MFG‐E8 in C2C12 cells by LC–MS/MS analysis. HSPA1L possesses a highly conserved domain structure crucial for protein stabilization, cell proliferation, and apoptosis, as well as cell signal transduction.^20^ HSPA1L forms a complex with lamp2 promotes chaperon‐mediated autophagy (CMA),^43^ and induces the translocation of Parkin to the damaged mitochondria via P62 inhibition. Since HSPA1L is an important interacting protein of Parkin,^20^ MFG‐E8 regulates mitophagy and could be realized through a novel signal pathway (MFG‐E8‐HSPA1L‐Parkin).

In conclusion, MFG‐E8 is a crucial protein that is targeted by DP and has a unique role in regulating mitophagy through the MFG‐E8‐HSPA1L‐Parkin signal pathway. DP down‐regulated the expression of MFG‐E8, reduced mitophagy deficiency, and alleviated the symptoms of diabetic sarcopenia, which could be considered a novel therapeutic strategy for diabetic sarcopenia.

## Conflict of interest

The authors declare no conflict of interest.

## Ethics statement

The animal use protocol listed below has been reviewed and approved by the animal ethics and welfare committee (AEWC) of Shandong University Cheeloo College of Medicine (Permit Number: 21170). Efforts were made to minimize the uneasiness of animals during all processes.

## Supporting information


**Table S1.** Report of LC–MS/MS.

**Figure S1.** Protocols and cell viability. (a) Pre‐incubation protocol. Cells were pre‐incubated with 160 μM DP for 4 hours before co‐incubation with 100 mg/L AGEs or 30 g/L DG. (b) The intervention protocols include Parts 1–3. (c) Diagram of C2C12 cells´ viability with different concentrations of DP, AGEs, and DG in 24 or 48 hours. (d) Cell viability of pre‐incubation protocol. Cell viability of AGEs and DG were statistically significantly decreased in comparison with CC (all *P* < 0.01). There is no statistical difference between the DP + AGEs (or DP + DG) and AGEs (or DG). However, there is a statistical difference between the DP → DP + AGEs (84.45 ± 2.27%) and AGEs (or DP + AGEs) (*P* < 0.001), and there is a statistical difference between the DP → DP + DG (82.79 ± 5.08%) and AGEs (or DP + DG) (*P* < 0.05). (*, *P* < 0.05; **, *P* < 0.01; ***, *P* < 0.001.).
**Figure S2.** The transfection efficiency images and quantitative analysis with qPCR and WB. (a) Transfection efficiency of Mover and MsiRNA in 24 and 48 hours. Scale bar = 200 μm. (b) Quantitative analysis of Mover and MsiRNA in 24 and 48 hours with Real‐time PCR assay. (c) WB strips of Mover and MsiRNA in 24 and 48 hours, and quantitative analysis.
**Figure S3.** Body weight change, and quantitative analysis of autophagosomes and cytolysosome in vitro. (a) Body weight change of 0w, 2w, 4w, 6w. (b) The quantitative analysis of autophagosome and cytolysosome. Autophagosome: The difference between STZ‐R1 (or P8) and R1 was statistically significant (all *P* < 0.01). The difference between STZ‐P8 (0.02 ± 0.01 per μm^2^) (or P8, 0.05 ± 0.01 per μm^2^) and DP + STZ‐P8 (0.07 ± 0.02 per μm^2^) was statistically significant (all *P* < 0.05). Cytolysosome: The difference between STZ‐R1 and R1 was statistically significant (*P* < 0.01). The difference between STZ‐P8 (0.02 ± 0.01 per μm^2^) and P8 (0.06 ± 0.02 per μm^2^) was statistically significant (*P* < 0.05). The difference between DP + STZ‐P8 (0.07 ± 0.03 per μm^2^) and STZ‐P8 (0.02 ± 0.01 per μm^2^) was statistically significant (*P* < 0.01). (*, *P* < 0.05; **, *P* < 0.01; ***, *P* < 0.001.).
**Figure S4.** The SA‐β‐gal staining images, WB strips, and quantitative analysis of P16 and P21 protein. (a) SA‐β‐gal staining images with Part 1 and Part 3 protocols in vitro. The SA‐β‐gal staining is darker in the AGEs, DG, and Mover groups, while the SA‐β‐gal staining is lighter after DP (or MsiRNA) intervention. Scale bar = 100 μm. (b) WB strips in part 1 protocol and quantitative analysis of P16 and P21 protein. (c) WB strips in part 3 protocol and quantitative analysis of P16 and P21 protein. (*, *P* < 0.05; **, *P* < 0.01; ***, *P* < 0.001.).
**Figure S5.** Images of mitophagy staining, WB strips of LC3B, P62, PINK1, and quantitative analysis in vitro. (a) Images of mitophagy staining in Part 2 protocol. Scale bar = 50 μm. Mitophagy area: The difference between 3‐MA (or Torin‐1) and CC was statistically significant (all *P* < 0.001). The difference between DP + 3‐MA (15.13 ± 1.91%) and 3‐MA (3.30 ± 0.50%), and the difference between Torin‐1 + AGEs (19.50 ± 1.12%)/DG (17.04 ± 2.24%) and Torin‐1 (27.63 ± 2.27%) was statistically significant (all *P* < 0.001). (b) WB strips in Part 2 protocol and quantitative analysis. (*, *P* < 0.05; **, *P* < 0.01; ***, *P* < 0.001.).
**Figure S6.** Images of JC‐1 staining in vitro. (a) Images of JC‐1 staining in Part 1 protocol. The green staining is darker and the red staining is weaker in AGEs and DG. After DP intervention, the dark green staining is alleviated in DP + AGEs and DP + DG groups. Scale bar = 20 μm. (b) Images of JC‐1 staining in Part 2 protocol. The green staining is darker and the red staining is weaker in 3‐MA. Torin‐1 alleviates the dark green staining and enhances the red staining. After DP intervention, the dark green staining is alleviated in DP + 3‐MA. After Torin‐1 intervention, the dark green staining is alleviated and the weak red staining is enhanced in the Torin‐1 + AGEs and Torin‐1 + DG. Scale bar = 20 μm. (c) Images of JC‐1 staining in Part 3 protocol. The green staining is darker and the red staining is weaker in Mover. MsiRNA alleviates the dark green staining, and enhances the weak red staining. After DP intervention, the dark green staining is alleviated in DP + Mover. After MsiRNA intervention, the dark green staining is alleviated and the weak red staining is enhanced in the MsiRNA +AGEs and MsiRNA +DG. Scale bar = 20 μm.
**Figure S7.** Flow cytometry and quantitative analysis. The flow cytometry diagram of mitochondrial membrane potential in Part 1–3 protocols. The quantitative analysis was evaluated by FL Red/Green intensity based on the results of the flow cytometry diagram, that is, (Q1 + Q2)/(Q2 + Q3). (a) Images of mitophagy staining in part 1 protocol. The difference between AGEs (DG, or DP) and CC was statistically significant (all *P* < 0.05). The difference between DP + AGEs (or DP + DG) and AGEs (or DG) was statistically significant (all *P* < 0.05), and the difference between DP + AGEs (or DP + DG) and DP was statistically significant (all *P* < 0.05). (b) Images of mitophagy staining in part 2 protocol. The difference between 3‐MA (Torin1, or DP + 3‐MA) and CC was statistically significant (all *P* < 0.05). Besides, the difference between Torin‐1 + AGEs (or Torin1 + DG) and Torin‐1 was statistically significant (all *P* < 0.001). (c) Images of mitophagy staining in part 3 protocol. The difference between Mover (or DP + Mover) and NC was statistically significant (all *P* < 0.001). Besides, the difference between MsiRNA+AGEs and MsiRNA was statistically significant (*P* < 0.05). (*, *P* < 0.05; **, *P* < 0.01; ***, *P* < 0.001.).
**Figure S8.** Immunofluorescence images of MFG‐E8 and Parkin, WB strips, and quantitative analysis in vitro. (a) Immunofluorescence images of Part 2 protocol. Scale bar = 100 μm. (b) WB strips in Part 2 protocol and quantitative analysis. (*, *P* < 0.05; **, *P* < 0.01; ***, *P* < 0.001.).
**Figure S9.** Full scan base peak MS chromatogram of the methanol extract of the sample.
