# Supplementary material for: The regulation of MFG‐E8 on the mitophagy in diabetic sarcopenia via the HSPA1L‐Parkin pathway and the effect of D‐pinitol
Source: J Cachexia Sarcopenia Muscle. 2024 Mar 29;15(3):934–48. doi: 10.1002/jcsm.13459 (PMC11154748; doi:10.1002/jcsm.13459)

**Supplement materials**

**The regulation of MFG-E8 on the mitophagy in diabetic sarcopenia via the HSPA1L-Parkin pathway and the effect of D-pinitol**

Wenqian Zhao^1,2,3^, Bin Zhao^4^, Xinyue Meng ^1,2,3^, Baoying Li^2,5^, Yajuan Wang^1,2,3^, Fei Yu^1,2,3^, Chunli Fu^1,2,3^, Xin Yu^1,2,3^, Xiaoli Li^6^, Chaochao Dai^2^, Jie Wang^2^, Haiqing Gao^1,2,3^, Mei Cheng^*1,2,3^

1. Materials and methods

1.1 CCK-8 assay

Cells were seeded at 2×10^3^ cells per well in 100μL culture medium in a 96-well plate. After cell adhesion, cells were exposed to various concentrations of AGEs, DG, and DP for a specific time. CCK-8 reagent (MCE, USA) was added to each well according to the manufacturer´s instructions and then incubated for another 2 hours. An absorbance microplate reader (Sunrise^TM^, TECAN, Männedorf, Switzerland) was used to measure the absorbance at 562nm. Cell viability and IC50 of AGEs (120mg/L) and DG (54g/L) for 48 hours were calculated by GraphPad Prism 5.0 for Windows (GraphPad Software, Inc., La Jolla, CA). After transfected with MFG-E8 plasmid (2.5, 3, or 3.5ng/ml) or MsiRNA (2, 2.5, or 3μl/ml), cell viability was not affected.

1.2 Reverse transcription and fluorescence quantitative PCR assay

10^5^ cells were seeded in each well of a 6-well plate. The cells were transfected with MFG-E8 DNA plasmid (3μg/ml) or siRNA (2.5μl/ml) after adhesion. After 6 hours, cells were changed to a complete medium and continued to culture for 24 or 48 hours. Total RNA was extracted using RNAiso Plus (Takara, Otsu, Japan) according to the manufacturer´s instructions. After quantitation, total RNA was reverse transcribed using M5 SuperFast plus qPCR RT kit (Mei5 Biotechnology, Co., Beijing, China) at 42˚C for 15min. Then qPCR was performed using the SYBR green method (cw0659, Cwbiotech, Jiangsu, China) in a fluorescence quantitative PCR instrument (LC96, Roche, Switzerland) with cycling conditions as follows: 95˚C, 600 s, subsequently 45 cycles of 95˚C for 10 s, 60˚C for 30s, and 72˚C for 30s. The primers (KeyGEN BioTECH Co., Ltd. Nanjing, China): alpha-actin, AAGTCCTGCAAGTGAACAAGC and AGGTGTGGTGCCAGATCTTC; MFG-E8, CACTGTGAAACCGGTTGTTCT and CTCCTTGTCTCCACCGCTTT. Alpha-actin was used as the reference gene and the relative expression of the MFG-E8 gene was calculated as 2^-ΔCt^, where ΔCt = Ct_MFG-E8_-Ct_α-actin_. The expression of MFG-E8 in experimental groups was calculated as 2^-ΔΔCt^, where ΔΔCt = ΔCt_exp_-ΔCt_nc/cc_.

1.3 Senescence associated β-galactosidase (SA-β-gal) staining

SA-β-gal staining was performed to evaluate the cells´ senescence. After completing the Part 1 and Part 3 protocols, cells were fixed and stained with the SA-β-gal dye (Solarbio Life Sciences, Co., Ltd, China) followed by incubating at 37℃ for half an hour avoiding CO_2_. Then, green staining reflecting the degree of cells´ senescence was observed under an optical microscope (Olympus, CKX53, Japan).

1.4 JC-1 staining and flow cytometry (FCM) assay

JC-1 staining and flow cytometry were used to assess the mitochondrial membrane potential (MMP). Cells were subjected to Part 1-3 protocols (Figure S1. b), and then JC-1 staining (Solarbio) was performed under the manufacturer´s instructions. Under a fluorescence microscope, red fluorescence reflects a higher MMP while green fluorescence on the contrary. MMP quantification was performed with FCM following the manufacturer´s instructions with BD flow cytometry (Accuri C6 plus, BD Biosciences, Oakland, USA). FSC was set with FITC, SSC was set with PE, and values of Red/Green were calculated to measure the levels of MMP.

1.5 LC-MS/MS analysis

The beads incubated with anti-MFG-E8, accompanied by MFG-E8 and MFGE-8´s interaction proteins, were transferred into SDT cleavage solution (4% SDS, 100mM DTT, 100mM TrisHCl), and boiled for 5 min, then added 200µL UA buffer (8M Urea, 150mM Tris-HCl, pH8.0) and centrifuged. Added IAA (50mM IAA in UA) and shaken for 1min, and stayed in the dark at room temperature for 30min, then centrifuged. Added 100µL UA buffer and centrifuged. Added 100µL NH_4_HCO_3_ buffer and centrifuged. Added 40 µg Trypsin buffer (6µg Trypsin in 40µL NH4HCO3 buffer) and shaken at 600 rpm for 1min, and stayed for 16-18 hours at 37°C. Enzymatic peptides were desalted with C18 StageTip and dried in a vacuum, then the peptide was redissolved with 0.1% FA, and the concentration was measured with OD280. Finally, the peptides were subjected to LC-MS analysis. The peptides received chromatographic separation with the nano flow rate Easy nLC 1200 system (Thermo Scientific). Then, the peptides were isolated and Data Dependent Acquisition (DDA) mass spectrometry analysis was performed by Q-Exactive HF-X mass spectrometer (Thermo Scientific).

2. Supplement figures

2.1 Figure S1. Protocols and cell viability.


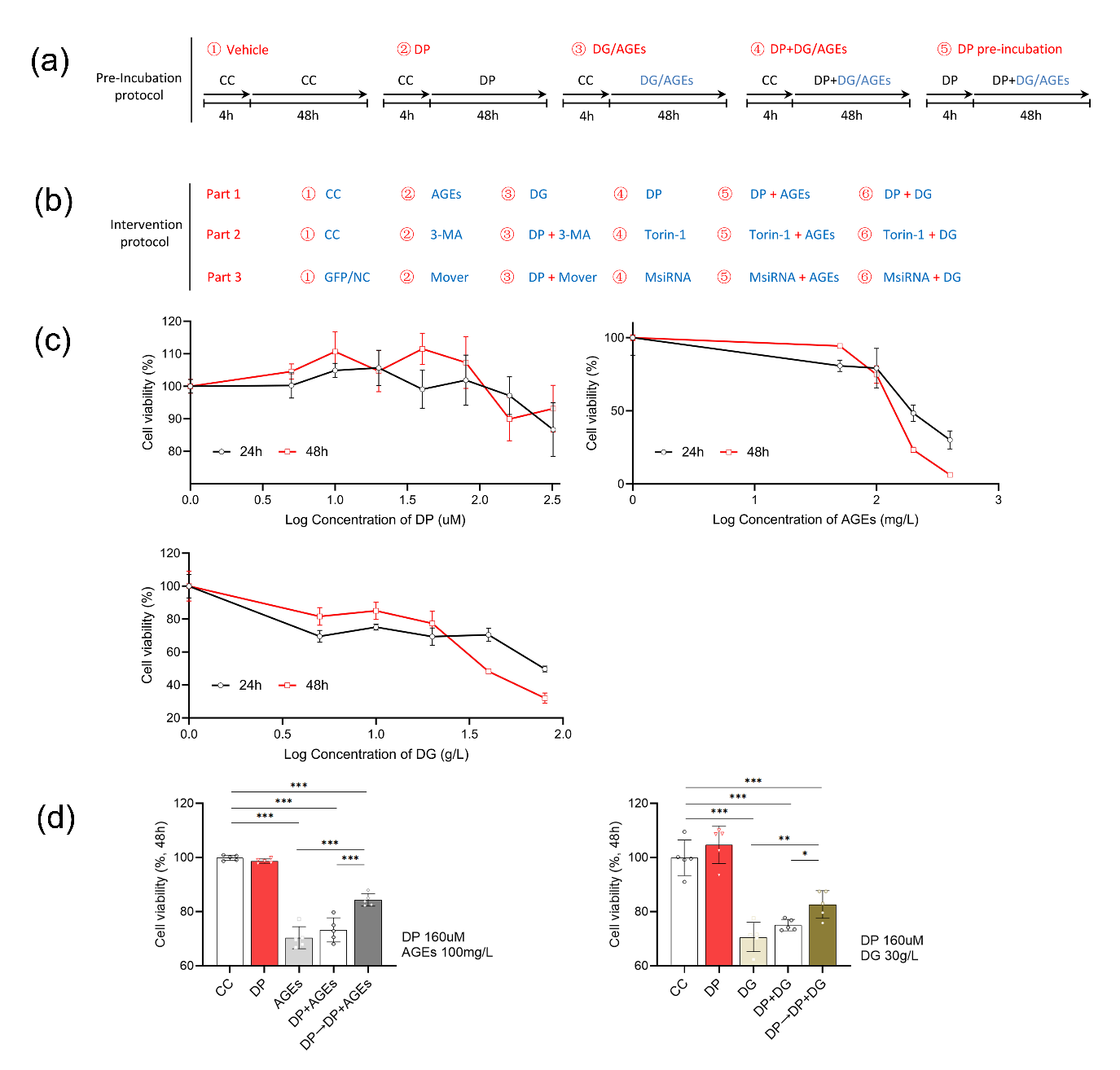


(a) Pre-incubation protocol. Cells were pre-incubated with 160μM DP for 4 hours before co-incubation with 100mg/L AGEs or 30g/L DG. (b) The intervention protocols include Parts 1-3. (c) Diagram of C2C12 cells´ viability with different concentrations of DP, AGEs, and DG in 24 or 48 hours. (d) Cell viability of pre-incubation protocol. Cell viability of AGEs and DG were statistically significantly decreased in comparison with CC (all *P*<0.01). There is no statistical difference between the DP+AGEs (or DP+DG) and AGEs (or DG). However, there is a statistical difference between the DP→DP+AGEs (84.45±2.27%) and AGEs (or DP+AGEs) (*P*<0.001), and there is a statistical difference between the DP→DP+DG (82.79±5.08%) and AGEs (or DP+DG) (*P*<0.05). (*, *P*<0.05; **, *P*<0.01; ***, *P*<0.001.)

2.2 Figure S2. The transfection efficiency images and quantitative analysis with qPCR and WB.


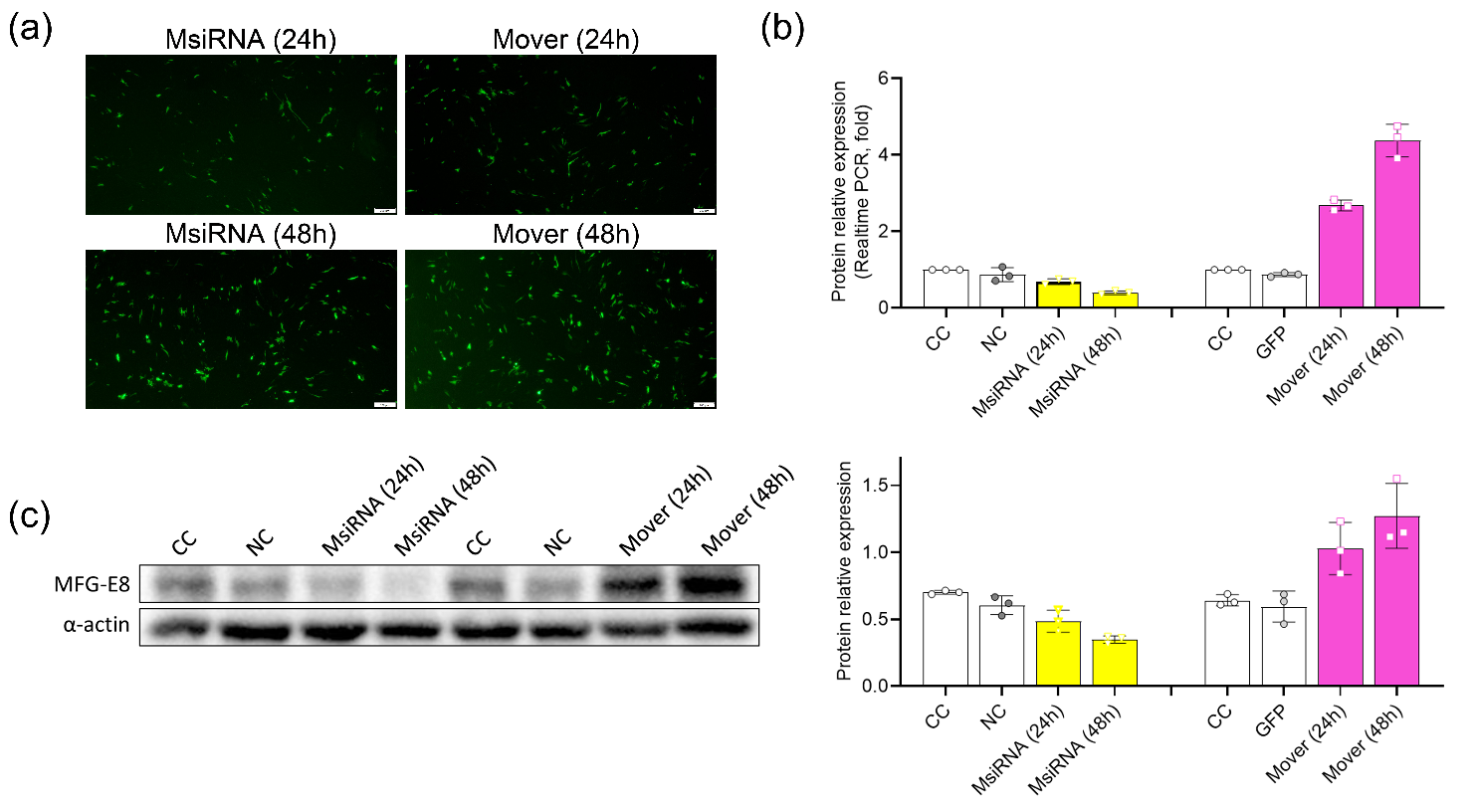


(a) Transfection efficiency of Mover and MsiRNA in 24 and 48 hours. Scale bar=200μm. (b) Quantitative analysis of Mover and MsiRNA in 24 and 48 hours with Real-time PCR assay. (c) WB strips of Mover and MsiRNA in 24 and 48 hours, and quantitative analysis.

2.3 Figure S3. Body weight change, and quantitative analysis of autophagosomes and cytolysosome in vitro.


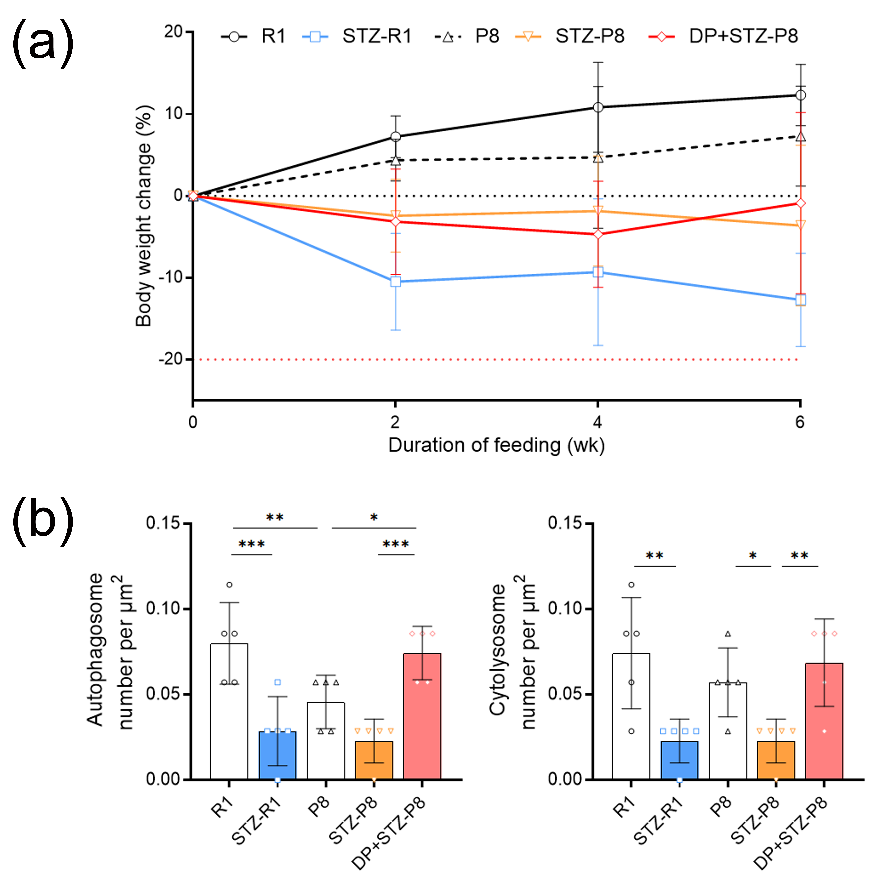


(a) Body weight change of 0w, 2w, 4w, 6w. (b) The quantitative analysis of autophagosome and cytolysosome. Autophagosome: The difference between STZ-R1 (or P8) and R1 was statistically significant (all *P*<0.01). The difference between STZ-P8 (0.02±0.01 per μm^2^) (or P8, 0.05±0.01 per μm^2^) and DP+STZ-P8 (0.07±0.02 per μm^2^) was statistically significant (all *P*<0.05). Cytolysosome: The difference between STZ-R1 and R1 was statistically significant (*P*<0.01). The difference between STZ-P8 (0.02±0.01 per μm^2^) and P8 (0.06±0.02 per μm^2^) was statistically significant (*P*<0.05). The difference between DP+STZ-P8 (0.07±0.03 per μm^2^) and STZ-P8 (0.02±0.01 per μm^2^) was statistically significant (*P*<0.01). (*, *P*<0.05; **, *P*<0.01; ***, *P*<0.001.)

2.4 Figure S4. The SA-β-gal staining images, WB strips, and quantitative analysis of P16 and P21 protein.


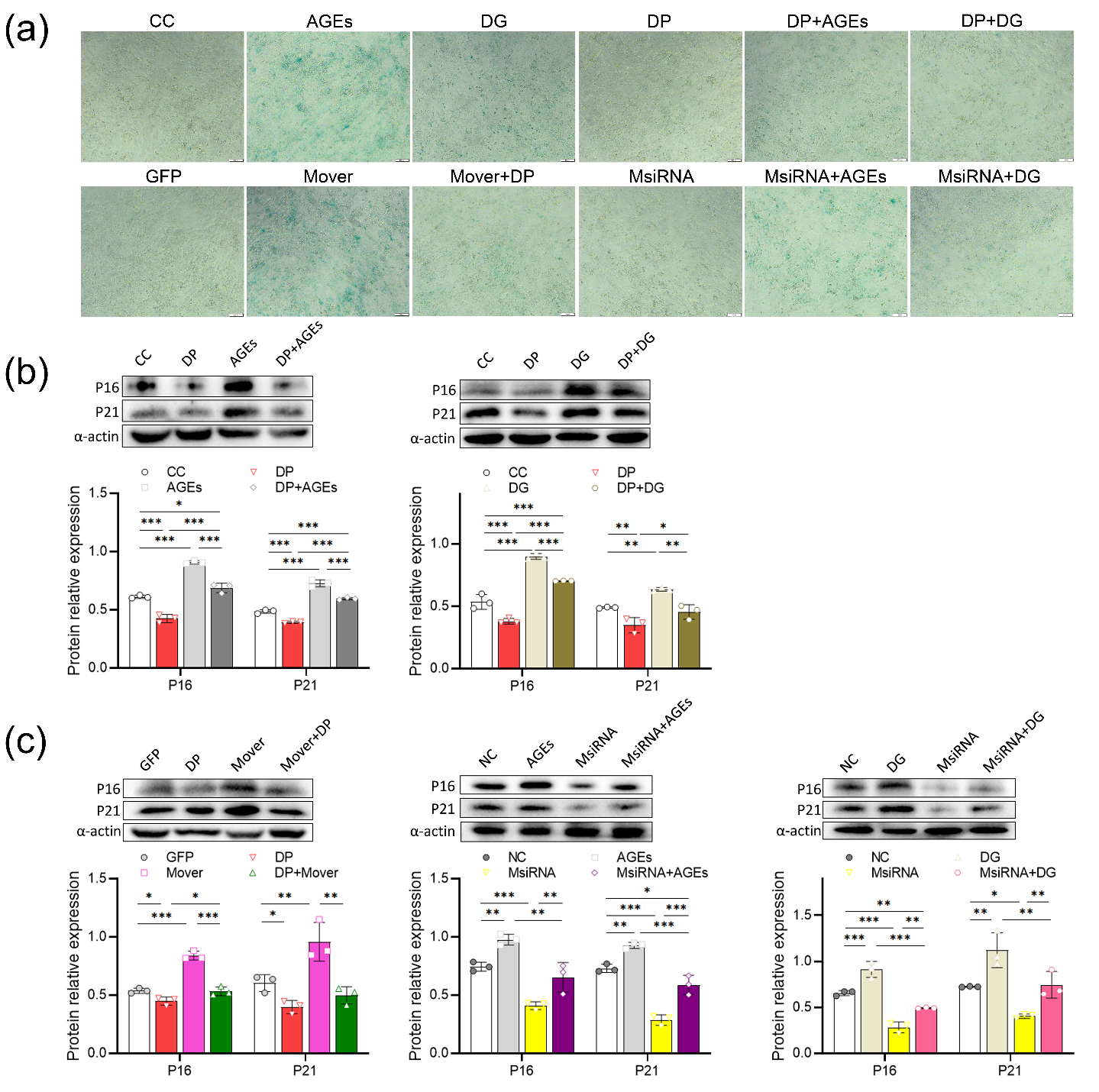


(a) SA-β-gal staining images with Part 1 and Part 3 protocols in vitro. The SA-β-gal staining is darker in the AGEs, DG, and Mover groups, while the SA-β-gal staining is lighter after DP (or MsiRNA) intervention. Scale bar=100μm. (b) WB strips in part 1 protocol and quantitative analysis of P16 and P21 protein. (c) WB strips in part 3 protocol and quantitative analysis of P16 and P21 protein. (*, *P*<0.05; **, *P*<0.01; ***, *P*<0.001.)

2.5 Figure S5. Images of mitophagy staining, WB strips of LC3B, P62, PINK1, and quantitative analysis in vitro.


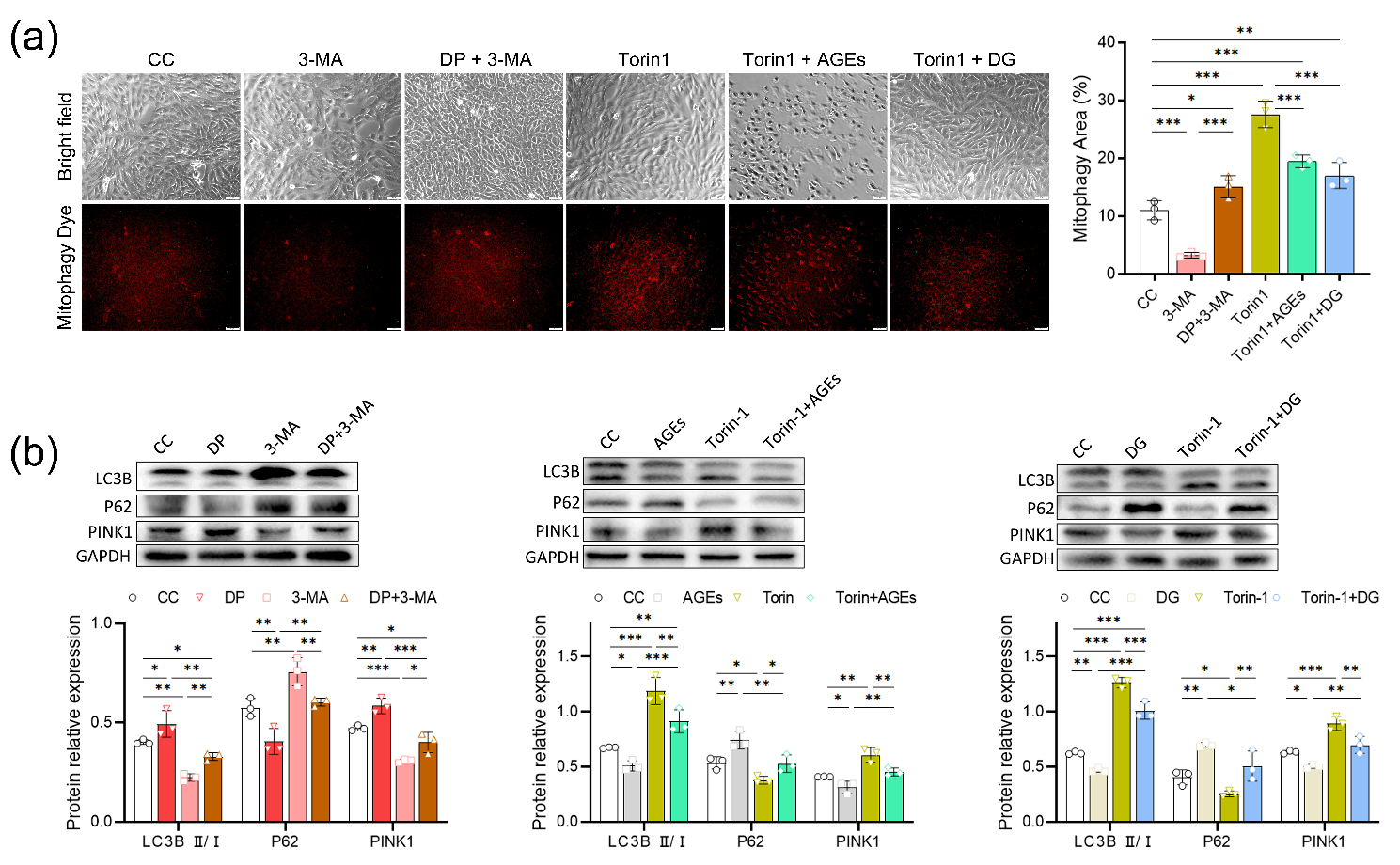


(a) Images of mitophagy staining in Part 2 protocol. Scale bar=50 μm. Mitophagy area: The difference between 3-MA (or Torin-1) and CC was statistically significant (all *P*<0.001). The difference between DP+3-MA (15.13±1.91%) and 3-MA (3.30±0.50%), and the difference between Torin-1+AGEs (19.50±1.12%)/DG (17.04±2.24%) and Torin-1 (27.63±2.27%) was statistically significant (all *P*<0.001). (b) WB strips in Part 2 protocol and quantitative analysis. (*, *P*<0.05; **, *P*<0.01; ***, *P*<0.001.)

2.6 Figure S6. Images of JC-1 staining in vitro.


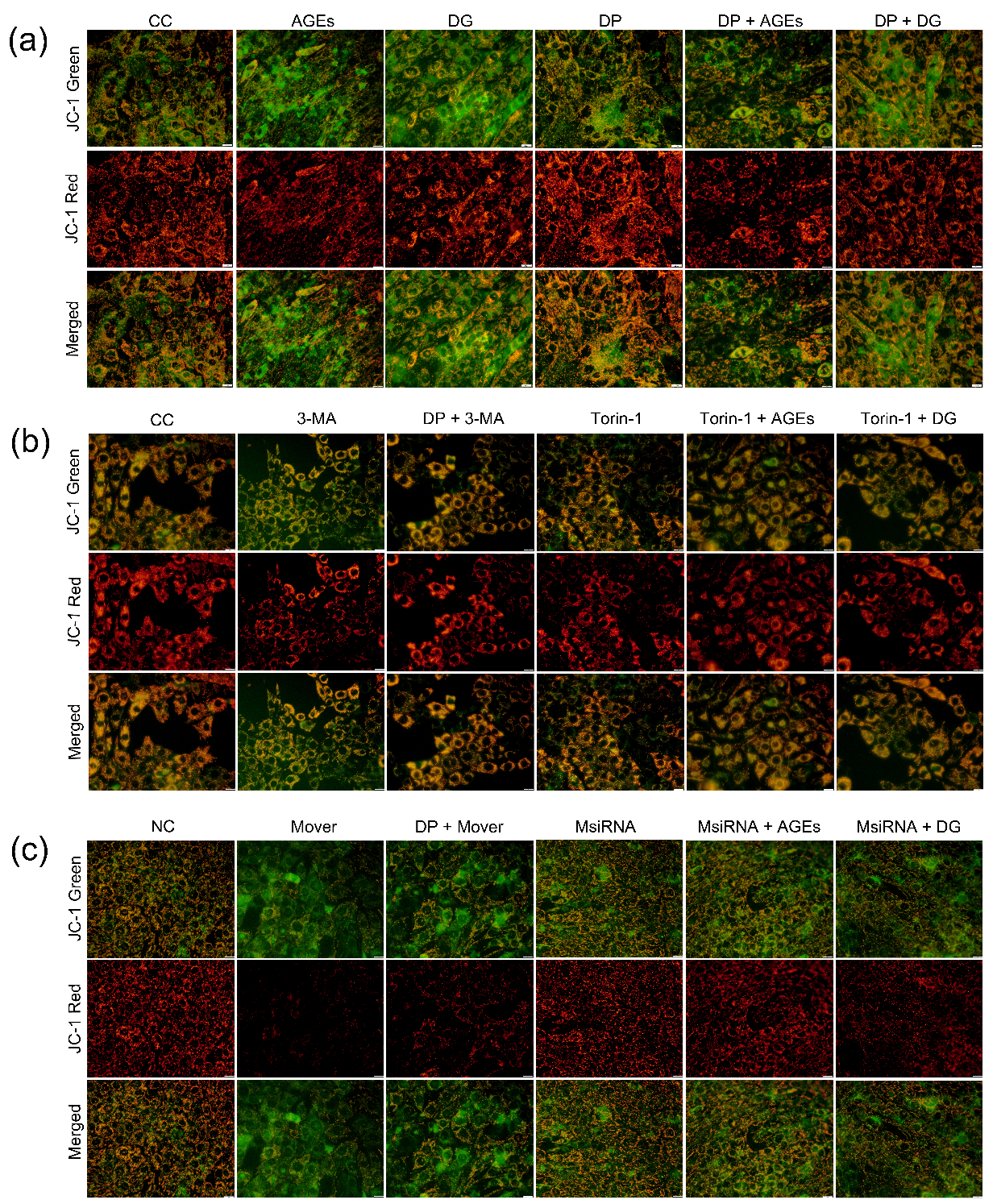


(a) Images of JC-1 staining in Part 1 protocol. The green staining is darker and the red staining is weaker in AGEs and DG. After DP intervention, the dark green staining is alleviated in DP+AGEs and DP+DG groups. Scale bar=20μm. (b) Images of JC-1 staining in Part 2 protocol. The green staining is darker and the red staining is weaker in 3-MA. Torin-1 alleviates the dark green staining and enhances the red staining. After DP intervention, the dark green staining is alleviated in DP+3-MA. After Torin-1 intervention, the dark green staining is alleviated and the weak red staining is enhanced in the Torin-1+AGEs and Torin-1+DG. Scale bar=20μm. (c) Images of JC-1 staining in Part 3 protocol. The green staining is darker and the red staining is weaker in Mover. MsiRNA alleviates the dark green staining, and enhances the weak red staining. After DP intervention, the dark green staining is alleviated in DP+Mover. After MsiRNA intervention, the dark green staining is alleviated and the weak red staining is enhanced in the MsiRNA +AGEs and MsiRNA +DG. Scale bar=20μm.

2.7 Figure S7. Flow cytometry and quantitative analysis.


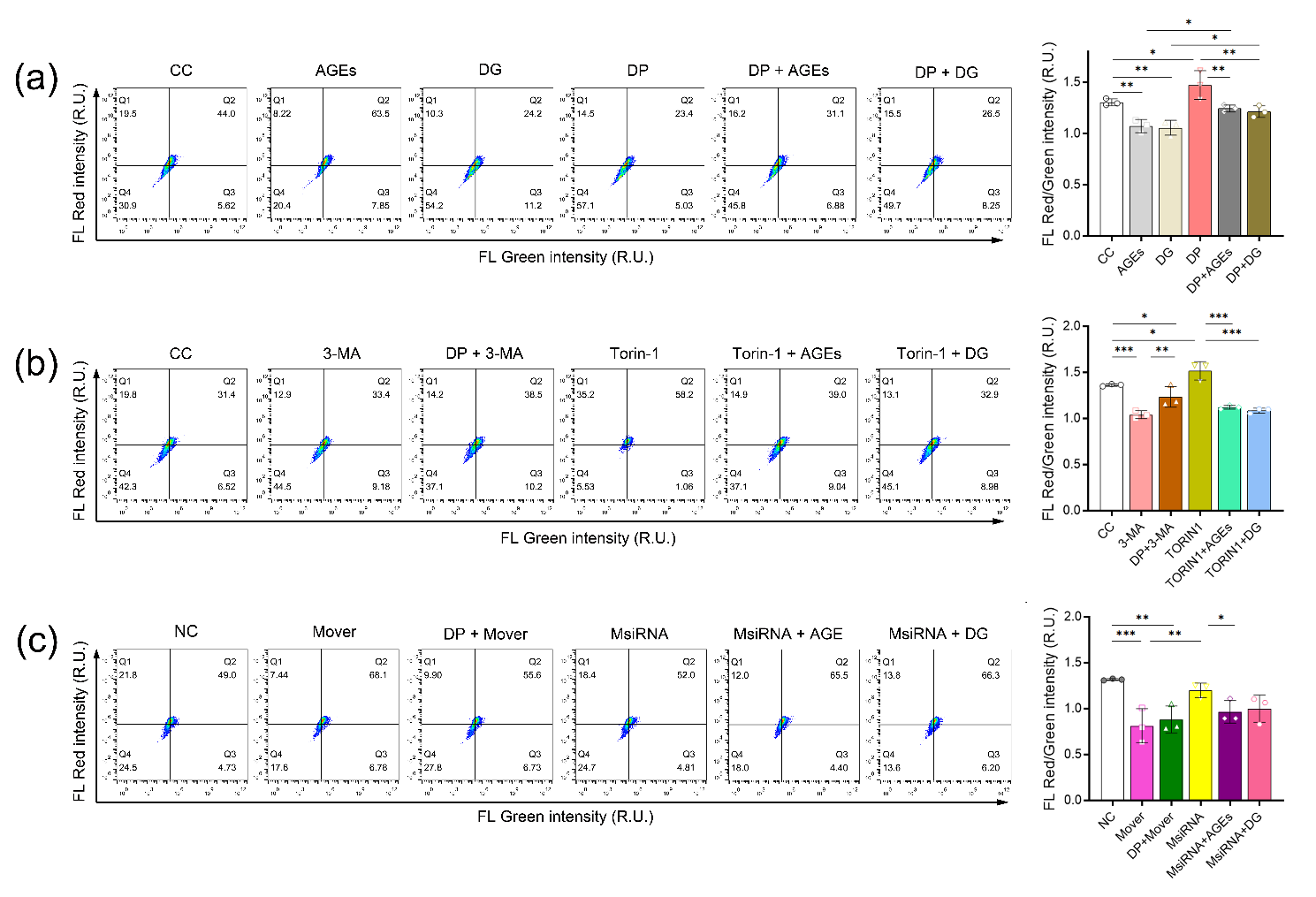


The flow cytometry diagram of mitochondrial membrane potential in Part 1-3 protocols. The quantitative analysis was evaluated by FL Red/Green intensity based on the results of the flow cytometry diagram, that is, (Q1+Q2)/(Q2+Q3). (a) Images of mitophagy staining in part 1 protocol. The difference between AGEs (DG, or DP) and CC was statistically significant (all *P*<0.05). The difference between DP+AGEs (or DP+DG) and AGEs (or DG) was statistically significant (all *P*<0.05), and the difference between DP+AGEs (or DP+DG) and DP was statistically significant (all *P*<0.05). (b) Images of mitophagy staining in part 2 protocol. The difference between 3-MA (Torin1, or DP+3-MA) and CC was statistically significant (all *P*<0.05). Besides, the difference between Torin-1+AGEs (or Torin1+DG) and Torin-1 was statistically significant (all *P*<0.001). (c) Images of mitophagy staining in part 3 protocol. The difference between Mover (or DP+Mover) and NC was statistically significant (all *P*<0.001). Besides, the difference between MsiRNA+AGEs and MsiRNA was statistically significant (*P*<0.05). (*, *P*<0.05; **, *P*<0.01; ***, *P*<0.001.)

2.8 Figure S8. Immunofluorescence images of MFG-E8 and Parkin, WB strips, and quantitative analysis in vitro.


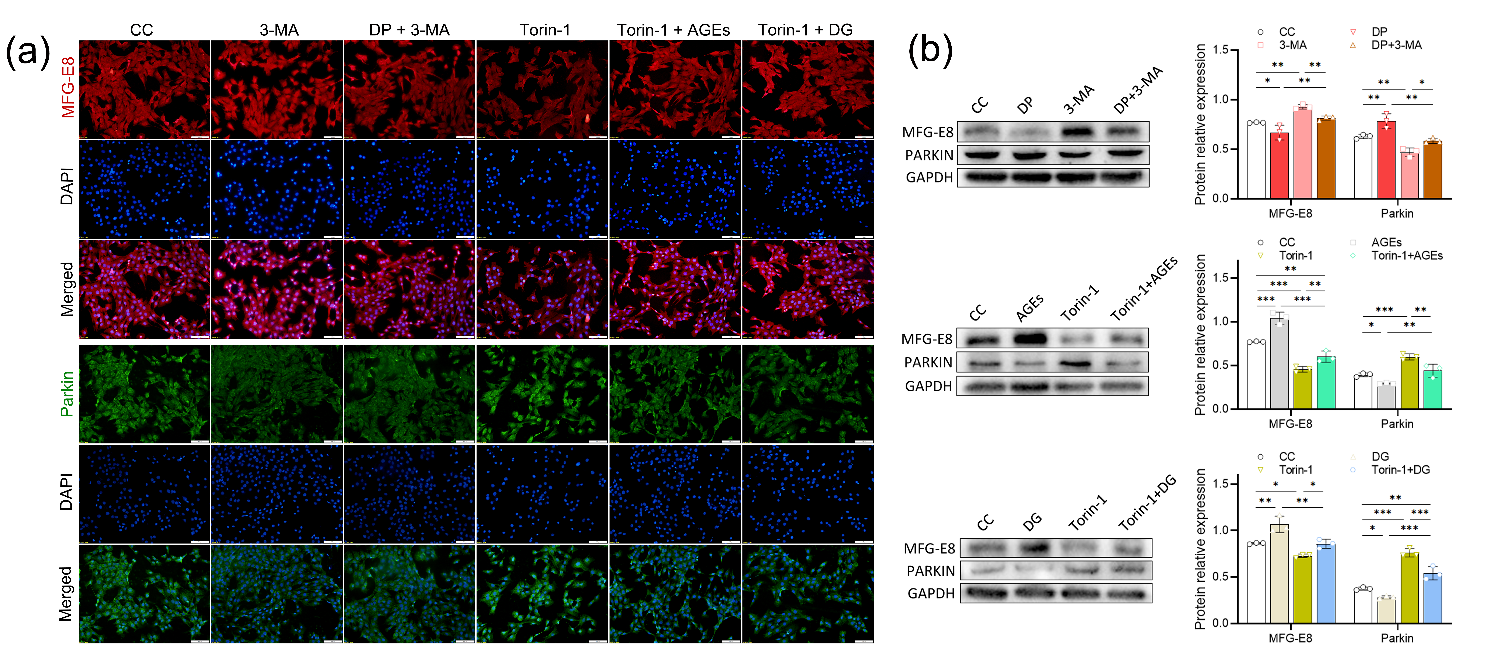


(a) Immunofluorescence images of Part 2 protocol. Scale bar=100μm. (b) WB strips in Part 2 protocol and quantitative analysis. (*, *P*<0.05; **, *P*<0.01; ***, *P*<0.001.)

2.9 Figure S9. Full scan base peak MS chromatogram of the methanol extract of the sample.


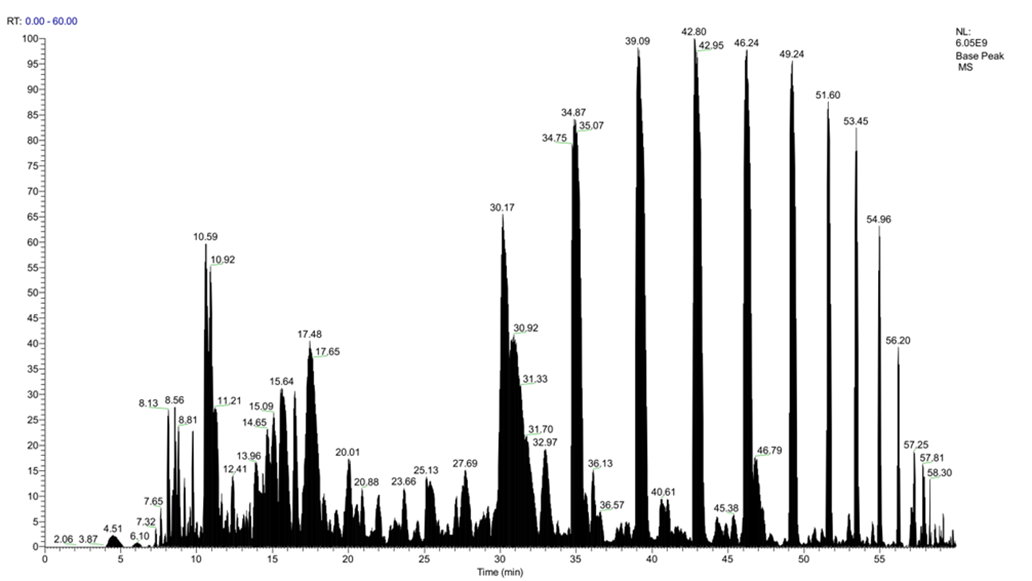

Supplement: Supplementary file 2 — Figure S1. Protocols and cell viability. (a) Pre‐incubation protocol. Cells were pre‐incubated with 160 μM DP for 4 hours before co‐incubation with 100 mg/L AGEs or 30 g/L DG. (b) The intervention protocols include Parts 1–3. (c) Diagram of C2C12 cells´ viability with different concentrations of DP, AGEs, and DG in 24 or 48 hours. (d) Cell viability of pre‐incubation protocol. Cell viability of AGEs and DG were statistically significantly decreased in comparison with CC (all P < 0.01). There is no statistical difference between the DP + AGEs (or DP + DG) and AGEs (or DG). However, there is a statistical difference between the DP → DP + AGEs (84.45 ± 2.27%) and AGEs (or DP + AGEs) (P < 0.001), and there is a statistical difference between the DP → DP + DG (82.79 ± 5.08%) and AGEs (or DP + DG) (P < 0.05). (*, P < 0.05; **, P < 0.01; ***, P < 0.001.). Figure S2. The transfection efficiency images and quantitative analysis with qPCR and WB. (a) Transfection efficiency of Mover and MsiRNA in 24 and 48 hours. Scale bar = 200 μm. (b) Quantitative analysis of Mover and MsiRNA in 24 and 48 hours with Real‐time PCR assay. (c) WB strips of Mover and MsiRNA in 24 and 48 hours, and quantitative analysis. Figure S3. Body weight change, and quantitative analysis of autophagosomes and cytolysosome in vitro. (a) Body weight change of 0w, 2w, 4w, 6w. (b) The quantitative analysis of autophagosome and cytolysosome. Autophagosome: The difference between STZ‐R1 (or P8) and R1 was statistically significant (all P < 0.01). The difference between STZ‐P8 (0.02 ± 0.01 per μm2) (or P8, 0.05 ± 0.01 per μm2) and DP + STZ‐P8 (0.07 ± 0.02 per μm2) was statistically significant (all P < 0.05). Cytolysosome: The difference between STZ‐R1 and R1 was statistically significant (P < 0.01). The difference between STZ‐P8 (0.02 ± 0.01 per μm2) and P8 (0.06 ± 0.02 per μm2) was statistically significant (P < 0.05). The difference between DP + STZ‐P8 (0.07 ± 0.03 per μm2) and STZ‐P8 (0.02 ± 0.01 per μm2) was [file JCSM-15-934-s001.docx]
